# Bidentate Substrate Binding Mode in Oxalate Decarboxylase

**DOI:** 10.3390/molecules29184414

**Published:** 2024-09-17

**Authors:** Alvaro Montoya, Megan Wisniewski, Justin L. Goodsell, Alexander Angerhofer

**Affiliations:** Department of Chemistry, University of Florida, P.O. Box 117200, Gainesville, FL 32611, USA

**Keywords:** oxalate decarboxylase, ENDOR, substrate binding, bidentate coordination

## Abstract

Oxalate decarboxylase is an Mn- and O_2_-dependent enzyme in the bicupin superfamily that catalyzes the redox-neutral disproportionation of the oxalate monoanion to form carbon dioxide and formate. Its best-studied isozyme is from *Bacillus subtilis* where it is stress-induced under low pH conditions. Current mechanistic schemes assume a monodentate binding mode of the substrate to the N-terminal active site Mn ion to make space for a presumed O_2_ molecule, despite the fact that oxalate generally prefers to bind bidentate to Mn. We report on X-band ^13^C-electron nuclear double resonance (ENDOR) experiments on ^13^C-labeled oxalate bound to the active-site Mn(II) in wild-type oxalate decarboxylase at high pH, the catalytically impaired W96F mutant enzyme at low pH, and Mn(II) in aqueous solution. The ENDOR spectra of these samples are practically identical, which shows that the substrate binds bidentate (κ*O*, κ*O*’) to the active site Mn(II) ion. Domain-based local pair natural orbital coupled cluster singles and doubles (DLPNO-CCSD) calculations of the expected ^13^C hyperfine coupling constants for bidentate bound oxalate predict ENDOR spectra in good agreement with the experiment, supporting bidentate bound substrate. Geometry optimization of a substrate-bound minimal active site model by density functional theory shows two possible substrate coordination geometries, bidentate and monodentate. The bidentate structure is energetically preferred by ~4.7 kcal/mol. Our results revise a long-standing hypothesis regarding substrate binding in the enzyme and suggest that dioxygen does not bind to the active site Mn ion after substrate binds. The results are in agreement with our recent mechanistic hypothesis of substrate activation via a long-range electron transfer process involving the C-terminal Mn ion.

## 1. Introduction

It is well known that transition metals are readily chelated by oxalate [1]. These complexes are sparingly soluble in water, and oxalate is predominantly found in bidentate coordination (κ*O*, κ*O*’) using two *cis*-positioned oxygen atoms to bind to the metal ion. The stability of these complexes in aqueous solution generally follows the Irving–Williams series [2], with Mn(II) showing a similar unimolecular stability constant as Ca ions [3]. Oxalate preferentially binds bidentate to both Mn(II) and Mn(III) in a predominantly octahedral geometry [4,5]. Owing to its symmetry, oxalate readily forms 1D coordination polymers as well as 2D and 3D networks where the oxalate di-anion functions as a bridge between neighboring Mn ions [5,6,7,8]. The tendency of oxalate to chelate Mn(II) is also evident in Mn-containing biomolecular complexes. All 19 X-ray crystal structures currently deposited in the Protein Data Bank (PDB) containing both protein-bound Mn and oxalate show bidentate binding between these co-factors (Supporting Information, Table S1 lists the PDB accession numbers, and Figure S5a–s provides images of their oxalate–Mn coordination geometry). Using ^17^O-labeled oxalate, EPR has been used to deduce bidentate coordination for the Mn–oxalate complex in pyruvate phosphate dikinase [9]. Both EPR and ^17^O-ENDOR (electron nuclear double resonance) were used to obtain a similar binding mode for the oxalate–ATP complex in pyruvate kinase [10,11,12].

Oxalate decarboxylase (OxDC) is an interesting Mn- and oxygen-dependent enzyme that catalyzes the heterolytic cleavage of the inert carbon–carbon bond of oxalic acid [13,14,15,16,17]. The enzyme employs redox chemistry despite the fact that the catalyzed reaction is nominally redox-neutral. In this protein, two Mn ions per single protein subunit are coordinated in two very similar β-barrel sites, so-called cupin domains [18], by three histidine and one glutamate residue [19,20]. The remaining ligand space is occupied by one or two water molecules, and if present in solution, small carboxylates like formate or acetate can bind to the N-terminal Mn(II) ion [19,20,21]. Both Mn ions are crucial for catalysis [22], and elucidating their structure and function is critical for a full understanding of the catalytic mechanism of OxDC.

Given the predominance of bidentate coordination between oxalate and Mn(II) in both proteins and model systems, it is surprising that substrate binding in OxDC has always been assumed to be monodentate in the relevant literature [16,23]. The N-terminal Mn-binding site is generally regarded as the active site with a flexible amino acid loop gating substrate access, while the C-terminal Mn-binding site is considered to be catalytically inactive without any indication of a solvent channel that would allow for substrate binding to this metal [20,24].

It has long been known that dioxygen is needed for OxDC enzymatic activity, which led to the assumption of an Mn(III)–superoxide complex as the driving force for catalysis [14]. If such a complex exists and is catalytically relevant, one would naturally assume that the substrate binds monodentate at the active site Mn ion since Mn prefers a hexa-coordinate environment. Yet, to date, there is no direct experimental evidence for dioxygen binding to Mn in OxDC nor the presence of an Mn(III)–superoxide complex. However, we recently identified a long-range electron transfer (LRET) pathway between the C- and the N-terminal Mn ions across subunit boundaries by way of a π-stacked tryptophan pair (W96/W274), which removes the necessity for dioxygen binding at the active-site and leaves the possibility open for bidentate binding of the substrate to a hexa-coordinate Mn ion [25]. As it turns out, there is enough space in the active site to accommodate a bidentate-bound substrate, assuming that the water ligands are removed from Mn and no residual dioxygen is located in the binding pocket [21,26].

On the other hand, support for a monodentate binding mode of the substrate comes from the crystal structure of the cobalt-substituted ΔE162 deletion mutant protein (Supporting Information, Table S2, and Figure S6b) [27]. Given that it does not contain Mn, it is inactive. The loss of E162 shortens the flexible SENST161–165 loop, which acts as a gate for substrate access, and it is therefore not clear whether this structure is representative of the active site of the wild-type (WT) enzyme. Moreover, kinetic isotope effect (KIE) measurements on WT OxDC suggest that mono-protonated oxalate is the substrate [28]. This suggests further that in the transition state, the substrate is monodentate coordinated to the Mn ion as it will be easier to protonate than a bidentate oxalate. However, one has to keep in mind that OxDC is still marginally active at pH values as high as 7 where the oxalate dianion is the predominant form in an aqueous solution [29]. Hence, the KIE results may perhaps also be explained by a deprotonation event prior to catalysis, i.e., during the formation of the enzyme–substrate complex.

The present contribution continues our long-standing interest in the catalytic mechanism of OxDC [25,30,31,32,33,34,35,36]. We report on ^13^C-ENDOR experiments designed to test for the binding mode of the substrate oxalate to the active site Mn(II) ion. Density functional theory (DFT) calculations, carried out on a reduced model of the active site, reveal both a mono- and a bidentate geometry for substrate binding, with the bidentate mode being energetically preferred.

For the ^13^C-ENDOR experiments, we used the W96F mutant enzyme of OxDC as a model for WT because its active site structure is identical to WT, but it shows strongly depressed kinetics [25]. This allows one to trap bound oxalate in the pre-transition state by rapid cooling after mixing the protein with the substrate. We also carried out ENDOR experiments on WT enzyme at high pH where only the dianion of oxalate is present, and no turnover occurs. This yields identical spectra. Based on the similarity of these ENDOR spectra with those of an aqueous Mn(II)–oxalate complex, we identify the binding mode of the substrate in OxDC as sideways (*cis*) bidentate (κ*O*, κ*O*’). This assignment is strongly supported by domain-based local pair natural orbital coupled cluster singles and doubles (DLPNO-CCSD) theory calculations of the expected ^13^C hyperfine coupling (hfc) constants and their comparison with the experiment. The simulated ENDOR spectra fit the experiment reasonably well for the case of the bidentate model and not at all for the monodentate one.

## 2. Results

### 2.1. ^13^C-ENDOR Spectra

**Figure 1:**
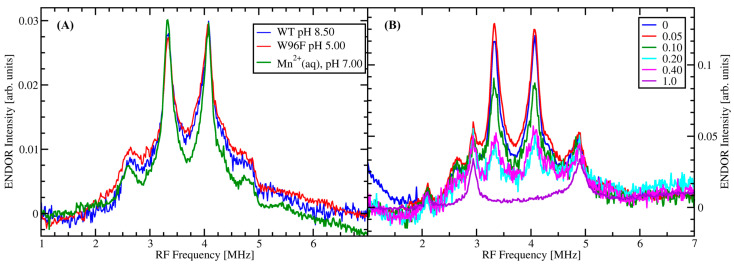
X-band ^13^C Mims ENDOR spectra. Experimental parameters: Microwave (MW) frequency = 9.746 GHz, MW pulse length (π/2) = 16 ns, τ = 540 ns, 521 points per spectrum. Radio frequency (RF) pulse width, *T* = 20 µs representing a π-pulse. The sample temperature was 5 K in all cases. (**A**) WT OxDC in Tris buffer pH 8.5 (blue), OxDC mutant W96F in citrate buffer pH 5.0 (red), and 1 mM MnCl_2_ aqueous solution (green). All samples contained 50 mM ^13^C-oxalate, and 50% glycerol as a glassing agent. The protein samples also contained 10 mM ascorbate and 5 μM DTPA. (**B**) X-band ^13^C Mims ENDOR spectra of 1 mM MnCl_2_ in 50:50 water/glycerol mixture with 50 mM ^13^C-oxalate, showing the loss of the ^13^C-ENDOR signal with increasing DTPA concentration. The molar ratio of DTPA:Mn(II) is given in the box, i.e., the DTPA concentrations are: (black) no DTPA, (red) 50 µM, (green) 100 µM, (cyan) 200 µM, (magenta) 400 µM, and (violet) 1000 µM.

Figure 1A depicts the ^13^C-ENDOR spectra of WT and OxDC mutant W96F at pH 8.5 and 5.0, respectively, as well as that of an aqueous solution of Mn(II) in the presence of ^13^C-labeled oxalate. It should be noted that we chose the pH of 8.5 for WT OxDC to exclude any interference from the residual activity of the enzyme at pH 7 [29]. The ^13^C-ENDOR signal is not observed in the absence of ^13^C-labeled oxalate (Supporting Information, Figure S2). The spectra were taken at 345 mT on the maximum of the echo-detected field sweep spectrum (Supporting Information, Figure S1), which mainly contains intensity from the |+½> ↔|−½> *m*_S_ EPR transition with relatively little contribution from transitions to higher spin manifolds. The ENDOR spectra are characterized by a distinct Pake pattern with two main peaks at 3.32 and 4.07 MHz, centered around the ^13^C Larmor frequency at 3.70 MHz. Shoulders appear symmetrical at lower and higher frequencies. All three spectra are strikingly similar to each other with respect to the positions of the peaks and shoulders.

Diethylenetriaminepentaacetic acid (DTPA) was added to the protein samples in order to chelate any Mn(II) that might have leached out of the protein during preparation to prevent it from forming a complex with the labeled oxalate. DTPA is a well-known hexadentate ligand for transition metal ions with a formation constant of log(*K*_f_) = 15.5 for Mn(II) [37]. On the other hand, the complex formation constants for oxalate complexes with Mn(II) are log(*K*_1_) = 2.65, log(*K*_2_) = 4.35, and log(*K*_3_) = 5.37 for complexation with one, two, and three oxalate dianions, respectively [3], which means that DTPA can be used to chelate adventitious free Mn(II) in the sample solution and prevent it from contributing to the ^13^C-ENDOR signal. This was tested by investigating aqueous 1 mM Mn(II) solutions in the presence of 50 mM ^13^C-labeled oxalate and increasing amounts of DTPA varying from 0 to 1 mM (Figure 1B). Clearly, with a concentration ratio between Mn(II) and DTPA of 1:1, the ^13^C-ENDOR signal is completely removed, indicating the displacement of oxalate by DTPA in complex formation with Mn. Since the addition of 5 μM DTPA to the protein solutions did not change their EPR or ^13^C-ENDOR signals, we conclude that the ENDOR signals shown in Figure 1A for the OxDC samples are from ^13^C-labeled oxalate coordinated to protein-bound Mn(II).

Please note that the signals at 2.94 and 4.89 MHz in Figure 1B are matrix proton ENDOR signals excited by the 5th and 3rd harmonics of the RF frequency, respectively. They appear when the RF amplifier is operated under saturation conditions. We can assume that the matrix protons are not affected by Mn(II) when it is complexed by either DTPA or oxalate. Therefore, these signals afford a simple internal intensity standard against which the ^13^C-ENDOR signals can be calibrated as long as the instrument settings and the Mn concentration in the samples stay the same. The data acquired on the protein samples in Figure 1A do not show these harmonics because the spectra were taken with the RF amplifier in its linear regime in order not to distort the ENDOR lines.

### 2.2. DFT Calculations

We simulated the substrate binding modes by geometry optimization with DFT using the program Gaussian, version 16 [38]. Details of the calculation are provided in Section 4. Our minimal model includes the N-terminal Mn(II) ion, its coordinating ligands, H95, H97, E101, H140, and the second shell ligands R92 and W132, both of which are known to play important roles in catalysis [20,39]. The substrate, mono-protonated oxalate, and a water molecule were also included in the model. The α carbons of the six amino acids were capped with methyl groups and frozen in space while all other atoms and the Mn ion were free to move. The coordinates for the starting structures were taken from our low-pH crystal structure, PDB ID 5VG3 [26], and protons were added using the program PyMOL [40,41]. The substrate was inserted using the molecular editor Avogadro [42].

**Figure 2:**
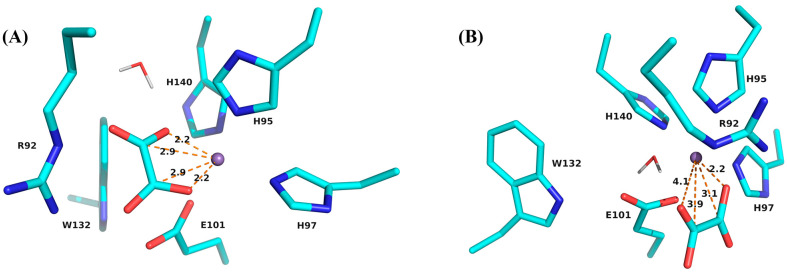
Results of geometry optimization of the substrate bound to the active site N-terminal Mn(II) ion. (**A**) Bidentate binding conformation. (**B**) Monodentate binding conformation. Distances between C and Mn, as well as coordinating O and Mn, are indicated by dashed lines and given in units of Ångstrom. Atom colors follow a modified CPK scheme where carbon atoms appear in cyan.

Geometry optimization results in two minima, a global minimum, with a bidentate substrate binding mode (Figure 2A) and a local minimum with a monodentate substrate binding mode (Figure 2B). Frequency calculations of these structures do not contain negative frequencies. The bidentate binding mode is approximately 4.7 kcal/mol lower in energy than the monodentate. The water molecule provides the sixth ligand for the octahedral coordination of the Mn(II) ion in the monodentate case while it moves away from the Mn ion when oxalate is bound bidentate. The structural model for these calculations includes 93 atoms from the six residues, the mono-protonated substrate, the water molecule, and the N-terminal Mn ion. This model is only slightly larger than our previous minimal model [21,43], and only provides a static picture of the active site. We consider the resulting models for oxalate coordination as initial estimates of the binding modes of the substrate. We are currently working on QM/MM calculations to explore the dynamic properties of the active site within the larger protein and its propensity to bind the substrate mono- or bidentate.

Our geometry-optimized models show distances between the carbons on the substrate and the Mn(II) ion that are similar to those found in published crystal structures of Mn(II)–oxalate complexes (Supporting Information, Figure S3a–l). They are useful as starting points for simulation and interpretation of the ^13^C ENDOR spectra.

### 2.3. Spectral Simulations

For the theoretical calculations of the expected ^13^C hfc constants at a sufficiently high level of theory, we focus on compound III by Lethbridge et al. [5] since it conveniently features only two oxalate molecules bound to Mn(II) in the unit cell, one in bidentate and the other in monodentate coordination (Supporting Information, Figure S3c). The remaining three ligands are hydroxides. This is a fairly compact model system with only 19 atoms (1 × Mn, 11 × O, 4 × C, and 3 × H), which allows for keeping computational costs reasonable.

**Figure 3:**
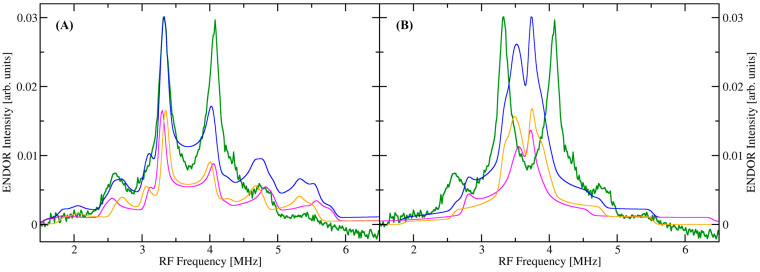
Simulations of the ^13^C ENDOR spectra using a bidentate (**A**) and a monodentate (**B**) model for substrate binding to Mn(II). Green: Experimental ENDOR spectrum. Pink and orange: individual contributions of C_1_ and C_2_ (**A**) or C_3_ and C_4_ (**B**) to the simulated ENDOR spectrum. Blue: sum of the individual contributions from the two carbon atoms. Supporting information, Figure S3c, shows the structure on which the calculations were based and the carbon numbering scheme.

We calculated the expected hfc constants using DLPNO-CCSD using the program ORCA [44,45,46]. This method is widely used to accelerate CCSD calculations by selectively summing only over the most significant electron pair correlation energies [47,48,49,50,51]. It lowers the cost of CCSD and CCSD(T) level accuracy to DFT level costs [52,53,54,55]. The method has proven reliable and very competitive compared to common DFT methods employed in the calculation of hfc parameters, particularly for transition metal ions [56,57,58,59,60]. Here, we follow the approach by Saitow and Neese [57] (see details in Section 4). The corresponding ^13^C ENDOR spectra were then simulated using the EasySpin toolbox for MATLAB™ [61]. The bidentate model gives results that compare quite well with the observed ENDOR spectra (Figure 3A). On the other hand, one can clearly see that the monodentate model with its larger C–Mn distances predicts smaller hfc constants for both the isotropic and the dipolar components (Table 1), which leads to a more crowded ENDOR spectrum near the free Larmor frequency of ^13^C (Figure 3B), which is not observed.

The two main peaks at 3.31 and 4.07 MHz that dominate the ENDOR spectra (Figure 1A) can be attributed to the perpendicular orientations of the corresponding ^13^C hyperfine tensors. The two ^13^C nuclei are not directly bonded to the Mn(II) in either binding pose, and their ENDOR spectra are therefore dominated by through-space electron–nuclear dipole coupling, which is described by the following equations where A_d_ is the dipolar component of the hfc tensor with *T* its principal component [62,63,64]. The values of the electronic and nuclear *g*-factors, magnetons *β_e_*_/*n*_, and the magnetic vacuum permeability *μ*_0_, are fundamental constants found in tables [65]. The electron spin density on Mn, *ρ*_eff_, can be approximated by unity [62], and *R* is the C–Mn distance measured in Å.

(1) $$A_{d}=\frac{\mu_{0}g\beta_{e}g_{n}\beta_{n}}{4\pi \;R^{3}}\left(\begin{array}{ccc} 2 & 0 & 0\\ 0 & -1 & 0\\ 0 & 0 & -1 \end{array}\right)=\left(\begin{array}{ccc} 2T & 0 & 0\\ 0 & -T & 0\\ 0 & 0 & -T \end{array}\right)$$

(2) $$T\approx \frac{19.86\cdot \rho_{\mathrm{eff}}}{R^{3}}\left(\mathrm{MHz}\right)$$

## 3. Discussion

The ^13^C-ENDOR spectra provide conclusive evidence that the substrate oxalate binds *cis* bidentate to the N-terminal Mn(II) ion in OxDC, i.e., in (κ*O*, κ*O*’) coordination. This conclusion can be inferred independent of any calculations because the ENDOR spectra taken with WT samples and aqueous Mn(II) at high pH are practically identical to those taken with the W96F mutant at low pH (Figure 1). Our choice of mutant (W96F) does not make a difference in the binding pose of oxalate since its active site structure is identical to that of the WT enzyme [25]. Our theoretical calculations also strongly support this conclusion (*vide infra*).

The DFT geometry optimization provides two different binding models for oxalate, bidentate and monodentate. The energy of the bidentate binding pose is approximately 4.7 kcal/mol lower than that of the monodentate one. While this also supports the bidentate binding mode as the energetically preferred state, we do not want to overinterpret this number, given that we worked with a relatively small active site model. The C–Mn distances in the bidentate model are of the order of 2.9 Å, which is near the lower range of distances from crystal structures of synthetic Mn–oxalate complexes (Supporting Information, Figure S3a–l) [5,66,67,68,69]. They also fall well within the range of distances observed in oxalate-coordinated Mn(II) in protein crystal structures (Supporting Information, Table S1 and Figure S5a–s). The monodentate model provides carbon–Mn distances of the order of 3.1 and 3.9 Å, also reasonably in line with what was observed in crystal structures of synthetic complexes [5] and the Co-substituted ΔE162 deletion mutant of OxDC [27]. The only other protein crystal structure containing Co coordinated with oxalate is that of Oxalate Biosynthetic Component A (ObcA) from *Burkholderia* (PDB ID 4NNC), where oxalate also binds monodentate but rather distantly (Supporting Information, Table S2 and Figure S6a) [70]. The binding poses of both models fit well within the static model of the active site cavity, which was obtained with the CAVER plugin for PyMOL (Supporting Information, Figure S4a,b).

Theoretical calculations of the ^13^C hfc constants and simulations of the corresponding ENDOR spectra were performed based on the structure of the two oxalate molecules in complex III from Lethbridge et al. (Supporting Information, Figure S3c) [5]. This structure was chosen because it contained both a mono- and a bidentate model of oxalate binding to Mn(II) with relatively few heavy atoms, an excellent option to minimize computational cost. We chose the DLPNO-CCSD level of theory instead of DFT for the hfc constants on the oxalate carbons because of its superior performance. This method is a local scaling approximation to the highly accurate but expensive coupled cluster singles and doubles method, which recovers 99.9% of the total correlation energy [50,51]. It has proven to be highly accurate for the calculation of both isotropic and anisotropic hyperfine coupling constants of open-shell systems, significantly beating the best DFT methods while only modestly increasing computational costs [56,57]. Table 1 shows the calculated hfc constants for the two carbons of the bidentate and monodentate oxalate model in the first row. From these numbers, the isotropic and dipolar parts were extracted and are shown in rows 2 and 3. Even though the oxalate carbons are not directly bound to Mn and are of the order of 3 Å away, their isotropic hfc cannot be neglected.

The remaining four rows in Table 1 show different structural and theoretical approximations to the C–Mn distances. The excellent agreement of the calculated ENDOR spectrum with the experiment suggests that the bidentate oxalate in complex III of Lethbridge et al. [5] represents the best structural approximation for both the aqueous complex and the protein. Oxalate is then expected to bind symmetrically in bidentate coordination (κ*O*, κ*O*’) with C–Mn distances of the order of 3.05 and 3.06 Å. If we take only the dipolar contribution of the calculated hfc tensors into account and recalculate the C–Mn distances with the point dipole model, these distances are overestimated by about 0.05 Å (row 4 in Table 1). On the other hand, our bidentate model from the DFT geometry optimization predicts a more tightly and more asymmetrically bound oxalate with C–Mn distances of the order of 2.95 and 2.86 Å. These values are still well within the range expected for bidentate oxalate in both model systems and protein (Supporting Information, Figures S3 and S5, and Table S1) but are probably less trustworthy given the small size of our active site model. Applying the point dipole model to just the main peaks of the ENDOR spectrum leads to similar C–Mn distances of 2.97 Å, which represents only a rough estimate due to the neglect of the isotropic hfc.

It was long assumed that the substrate for OxDC is the monoanion of oxalic acid because of its low second p*K*_a_ of 4.3. However, this argument is inconsistent with the observation that catalytic activity drops by only a factor of 13 when the pH is raised from 4.2 to 7.2 [29]. Additionally, it is important to note that the ^13^C-ENDOR spectra of oxalate bound to the OxDC mutant W96F at low pH are practically indistinguishable from that of oxalate bound to WT OxDC at pH 8.5 and Mn(II) in solution at pH 7 (Figure 1). This suggests that the species observed in all three cases is the same, i.e., the dianion of oxalate, C_2_O_4_^2−^. On the other hand, KIE measurements by Reinhardt et al. suggest that a deprotonation of the substrate takes place before decarboxylation, arguing for the monoanion as the substrate [28]. The fact that our data provides clear evidence for bidentate binding of the substrate does not invalidate the KIE data. In order to explain the apparent discrepancy, we want to point out that catalysis requires Mn(III) [35,71], and the KIE data either refers to an Mn(III)-bound oxalate species or a pre-transition state complex responsible for the rate-limiting step. Here, we observe an Mn(II)-bound oxalate species. Hence, our experiments likely probe a stable pre-catalytic enzyme–substrate complex that still needs to be activated. It is quite possible that our ENDOR experiments on Mn(II) do not probe the rate-limiting state responsible for the observed KIE. Another possibility is that the KIE experiments probe a rate-limiting deprotonation step before oxalate binds to Mn(II).

An important consequence of our results is that dioxygen cannot bind to Mn when the substrate is bound. Of course, this assumes that Mn(II) in the active site maintains an approximate hexa-coordinate geometry and that large-scale motions of any of its amino acid ligands are impossible since the active site is well buried within the bulk of the protein. As already stated in Section 1, there is no experimental evidence to date that dioxygen binds to the active-site Mn, nor is there evidence for an Mn–superoxide complex. Yet, Mn needs to be activated to Mn(III) for catalysis to proceed [35,71]. In our current view of the mechanism, there is no need for dioxygen to bind to the active site at any time. The oxidation of Mn(II) to Mn(III) happens via LRET from the C-terminal Mn ion after the substrate is bound [25,36]. This LRET is suppressed in the W96F mutant enzyme since W96 acts as a critical electron transfer bridge in conjunction with W274 [25]. In WT at pH 8.5, the reaction is completely suppressed.

## 4. Materials and Methods

Enzyme Preparation: All chemicals were purchased from Thermo Fisher Scientific (Walton, MA, USA) unless otherwise specified. OxDC and mutant enzyme containing a C-terminal His_6_ tag were purified using established protocols from the literature [14,25,35]. Site-directed mutagenesis for W96F was described by Pastore et al. [25]. Overnight cell cultures were grown in the presence of 50 mg/mL ampicillin in Luria–Bertani (LB) broth at 37 °C. Large (1 L) cell cultures were inoculated the following day with cells from the overnight culture and grown to an optical density of 0.5 at 600 nm. Cells were then heat-shocked at 42 °C for 15 min. Induction was performed by addition of 1 M MnCl_2_ to growth media for a final concentration of 4.6 mM and isopropyl β-D-1-thiogalactopyranoside (IPTG) for a final concentration of 0.8 mM. Cells were grown for 4 more hours before being harvested by centrifugation at 6000 revolutions per minute for 18 min at 4 °C. Cell pellets were stored at −80 °C until further use.

Cell pellets were resuspended in 40 mL of lysis buffer (50 mM Tris, 500 mM NaCl, 10 mM imidazole at pH 7.5) and lysed by high-power sonification. Lysate was incubated with nickel nitrilotriacetic acid (Ni:NTA) resin for two hours at 4 °C under gentle stirring. Ni:NTA resin was washed with 8 column volumes of wash buffer (20 mM Tris, 500 mM NaCl, 20 mM imidazole at pH 8.5). OxDC was collected from fractions as resin was washed with 6 column volumes of elution buffer (20 mM Tris, 500 mM NaCl, 250 mM imidazole, at pH 8.5) for a total volume of 30 mL. Imidazole was then removed by dialysis with storage buffer (50 mM Tris and 500 mM NaCl) at pH 8.5. Chelex 100 resin from Bio-Rad (Hercules, CA, USA) was used to remove dissolved free metal cations in solution. OxDC was concentrated using Amicon filters with a 30 kDa molecular weight cutoff. Protein concentration was determined with the Bradford assay [72] (Pierce, Rockford, IL, USA). Aliquots of OxDC (200–300 μL, containing 1.5 mg protein) diluted to 1.5 mL with Chelex treated storage buffer, followed by dilution to 10 mL with de-ionized water, and the addition of 200 µL of trace metal grade nitric acid were sent to the University of Georgia (Center for Applied Isotope Studies) for ICP-MS analysis of metal content, following previously established protocols [22]. Post-purified enzyme was then subjected to further dialysis steps for replacement of the Tris storage buffer. Enzyme solution was initially dialyzed into a poly-buffer (50 mM Tris, 50 mM Bis-Tris, 50 mM piperazine, 50 mM sodium citrate, and 500 mM NaCl in 50:50 water to glycerol) at pH 8.5, followed by lowering of the pH by 1 unit every 2 h with fresh buffer until the desired pH was achieved. Glycerol was added to the dialysis buffer in order to maintain stability for the enzyme. Attempts without glycerol typically result in the enzyme crashing out of solution near the isoelectric point, which is between pH 5 and pH 6. It is possible to dialyze the enzyme with less glycerol (20% *v*/*v*). However, a 50% *v*/*v* solution will give a glass upon freezing in liquid helium and yield a much higher signal/noise ratio in our pulsed EPR experiments. Therefore, all experiments were performed with a 1:1 glycerol:water ratio. Extensive dialysis is also performed at the desired pH (5.0) by moving from poly buffer to the final buffer solution (citrate buffer). The pH of the samples was checked after dialysis to confirm the pH of the solution. Adjustment of the buffer pH was performed by titration with concentrated HCl(*aq*) and KOH(*aq*) before addition of glycerol. To further slow down catalysis at low pH, 10 mM ascorbate was added to the W96F sample which effectively reduces any Mn(III) to Mn(II) [35]. For WT at pH 8.5, no dialysis was necessary and the enzyme was used in its Tris storage buffer.

Simulations and Calculations: The space in the active site around the N-terminal Mn ion was explored using the program CAVER (plugin version 3.0 for PyMOL) [73,74,75], which allows analysis and visualization of static solvent channels in crystal structures and interfaces directly with the visualization software PyMOL (version 2.5.1) [40,41]. A minimum sphere radius of 0.7 Å was chosen in order to size the static channel in the low pH crystal structure, PDB ID 5VG3, using the “open” configuration, where E162 does not occlude the accessibility of the active site [26]. Upon identification of the access channel and internal cavity boundaries, DFT optimizations of monoprotonated oxalate as the native substrate were performed in both monodentate and bidentate binding modes. Both modes were demonstrated to fit inside the available space, suggesting that monodentate binding is not the only possible orientation (Supporting Information, Figure S4a,b).

Geometry optimization of the two binding modes of oxalate was carried out with the Gaussian16 computational package [38]. Calculations were carried out with a minimal set of amino acid residues which was chosen to resemble the active site binding cavity. It included the four Mn-coordinating residues H95, H97, E101, and H140, as well as the second shell residues R92 and W132, a water molecule, and monoprotonated oxalate. The total charge of the assembly was zero. To model the mono- and bidentate binding modes of the substrate, the structures of metal-bound oxalate from crystal structures of the cobalt-substituted ΔE162 mutant OxDC [27] and a manganese-containing protein (TM1287) from *Thermotoga maritima* [76] were used as initial guesses. Oxalate binding to the Mn in the active site was then optimized using the auto-optimization tool with the UFF force field in Avogadro (version 1.2.0) [42] while keeping all other atoms frozen. In the case of the monodentate binding mode, the Mn-bound water molecule was additionally optimized with the auto-optimization tool in Avogadro using the UFF force field. These structures were used as the starting points for geometry optimization with DFT.

All optimizations were performed in the gas phase. Protein backbone atoms were removed and only R-groups were considered. The α carbon atoms were frozen in place in order to restrict large-scale deviations from the crystal structure and better simulate the local environment of the metal. High-spin multiplicities were used in all cases since they are most commonly observed for manganese and fit with experimentally observed states. DFT calculations were performed using the CAM-B3LYP57 functional [77] and the cc-pvdz59 basis set [78]. Frequency calculations were performed in all cases to verify energetic minima were reached using “VeryTight” convergence criteria and “UltraFine” integration grids. “VeryTight” criteria adds additional restrictions for the cutoffs on forces and step size that determine convergence. Use of “UltraFine” grids increases the number of points in the integration grid and is recommended for large systems with “soft modes” such as methyl rotations.

The theoretically expected hyperfine coupling constants were calculated using the DLPNO-CCSD method as implemented in ORCA (version 5.0.4) [44,45]. In order to keep computational cost at a reasonable level, we focused on complex III from Lethbridge et al. [5], which features a bidentate and a monodentate coordinated Mn(II) ion. The crystal structure data were used without further geometry optimization. Following Saitow and Neese [57], the second-order Douglas–Kroll–Hess (DKH) scalar relativistic Hamiltonian was used in combination with the DKH-recontracted def2-TZVPP basis set, which was shown to be successful in calculating the hyperfine constants of first-row transition metal complexes.

Predicted ENDOR spectra for the two substrate geometries were simulated with the output parameters from ORCA using the “salt” function in the EasySpin toolbox (version 5.2.4) for MATLAB™ (version R2022b, update 9) [61,79]. When simulating the spectra, the Mn(II) zero-field splitting parameters *D* and *E* were set to 600 and 100 MHz, respectively, since these values provided reasonable simulations of the EPR spectra (Supporting Information, Figure S1).

## 5. Conclusions

^13^C-ENDOR experiments show that oxalate binds sideways bidentate (κ*O*, κ*O*’) to the Mn(II) ion in the active site of the OxDC mutant W96F at low pH. The W96F mutant has the same active site structure as WT and therefore serves as a faithful model of WT OxDC [25]. In fact, our experiments also show bidentate binding of oxalate in WT OxDC at high pH where the enzyme is inactive. This has implications for the enzymatic mechanism since it prevents dioxygen from binding to the same Mn(II) ion as is often assumed in the older literature [16]. While our experiments do not probe the transition state nor necessarily the pre-transition state of the enzyme–substrate complex, both of which involve Mn(III), we can confidently state that the substrate binds sideways bidentate to Mn(II) in the active site in a (κ*O*, κ*O*’) coordination. In order to become catalytically active, Mn(II) has to be oxidized to Mn(III), which requires dioxygen or another one-electron oxidant [35]. The bidentate binding model for the substrate is compatible with our current hypothesis for the catalytic mechanism, i.e., that dioxygen binds to the C-terminal Mn ion instead of the N-terminal one. Electron transfer takes place from the N-terminal Mn via LRET after the substrate is already bound [25,36].

## Figures and Tables

**Table 1:** Calculated hyperfine coupling constants from DLPNO-CCSD calculations (using the structure of compound III from Lethbridge et al. [5], see Supporting Information Figure S3c for carbon numbering) and simulated vs. calculated C–Mn distances for bidentate and monodentate binding models of oxalate.

	Bidentate		Monodentate	
	C_1_	C_2_	C_3_	C_4_
*A* CCSD [MHz]	−0.7517, −0.8647, 1.1738	−0.6525, −0.7583, 1.2959	0.0412, 0.0815, 1.8179	0.0742, 0.0811, 0.7358
*A*_iso_ [MHz]	−0.1475	−0.0383	0.6469	0.2970
*A*_dip_ [MHz]	0.6607	0.6671	0.5855	0.2194
C–Mn distance ^†^ [Å]	3.117	3.107	3.245	4.501
C–Mn distance ^‡^ [Å]	3.060	3.053	3.260	4.569
C–Mn distance * [Å]	2.950	2.855	3.087	3.940
C–Mn distance ° [Å]	2.97	2.97		

^†^ Based on the point dipole approximation using the calculated *A*_dip_. ^‡^ Crystal structure data by Lethbridge et al. [5]. * From geometry optimization of oxalate in the active site of OxDC by DFT (Figure 2). ° Based on the main peaks of the ^13^C ENDOR spectrum (4.07 and 3.31 MHz), assuming a simple point dipole model.

## Data Availability

The original contributions presented in the study are included in the article/Supplementary Materials, further inquiries can be directed to the corresponding author.

## Supplementary Materials

The following supporting information can be downloaded at: https://www.mdpi.com/article/10.3390/molecules29184414/s1, Figure S1: Echo-detected field sweep spectra; Figure S2: ENDOR spectra of W96F at low pH with and without ^13^C-labeled oxalate; Figure S3a–l: Structures of model compounds of oxalate bound to Mn; Figure S4a,b: Results of Caver calculations [73]; Figure S5a–s: Protein structures in the PDB containing oxalate bound to Mn; Figure S6a,b: Protein structures in the PDB containing oxalate bound to Co; Table S1: List of proteins containing Mn-bound oxalate; Table S2: List of proteins containing Co-bound oxalate. References [80,81,82,83,84,85,86,87,88,89,90,91,92,93,94,95] are cited in the Supplementary Materials.
