# Supplementary material for: Bidentate Substrate Binding Mode in Oxalate Decarboxylase
Source: Molecules. 2024 Sep 17;29(18):4414. doi: 10.3390/molecules29184414 (PMC11433825; doi:10.3390/molecules29184414)
Supplement: Supplementary file 1 [file molecules-29-04414-s001.zip › molecules-3177211-supplementary.pdf]

Bidentate Substrate Binding Mode in Oxalate Decarboxylase – Supplementary Information

Alvaro Montoya, Megan Wisniewski, Justin L. Goodsell, and Alexander Angerhofer
Department of Chemistry, University of Florida, Box 117200, Gainesville, FL 32611

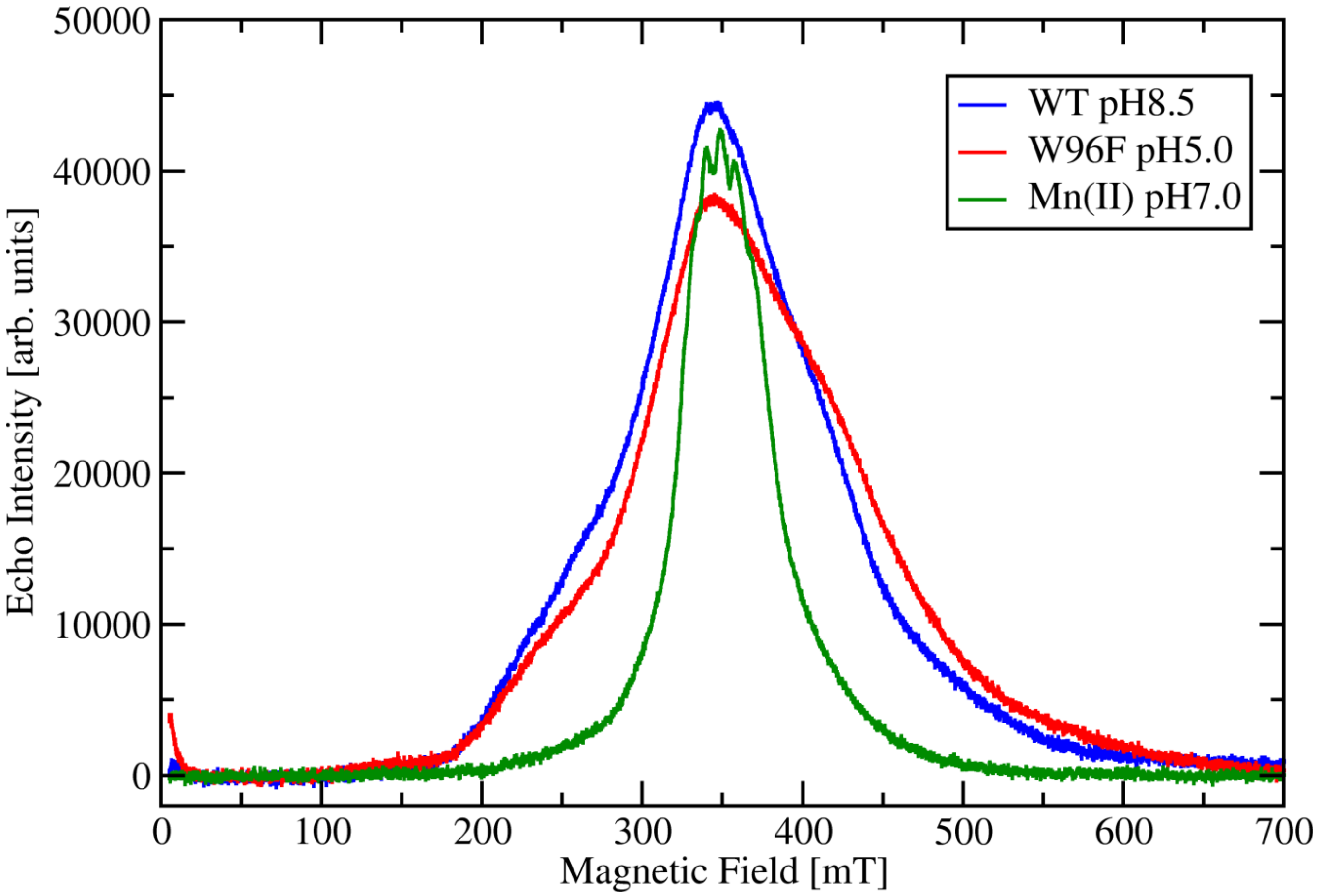

Figure S1: Echo-detected field sweep spectra taken at 5.2 K using the Hahn echo sequence. The width of the π/2 pulse was 16 ns and for the π pulse it was 32 ns. The delay time between the two pulses was 200 ns.

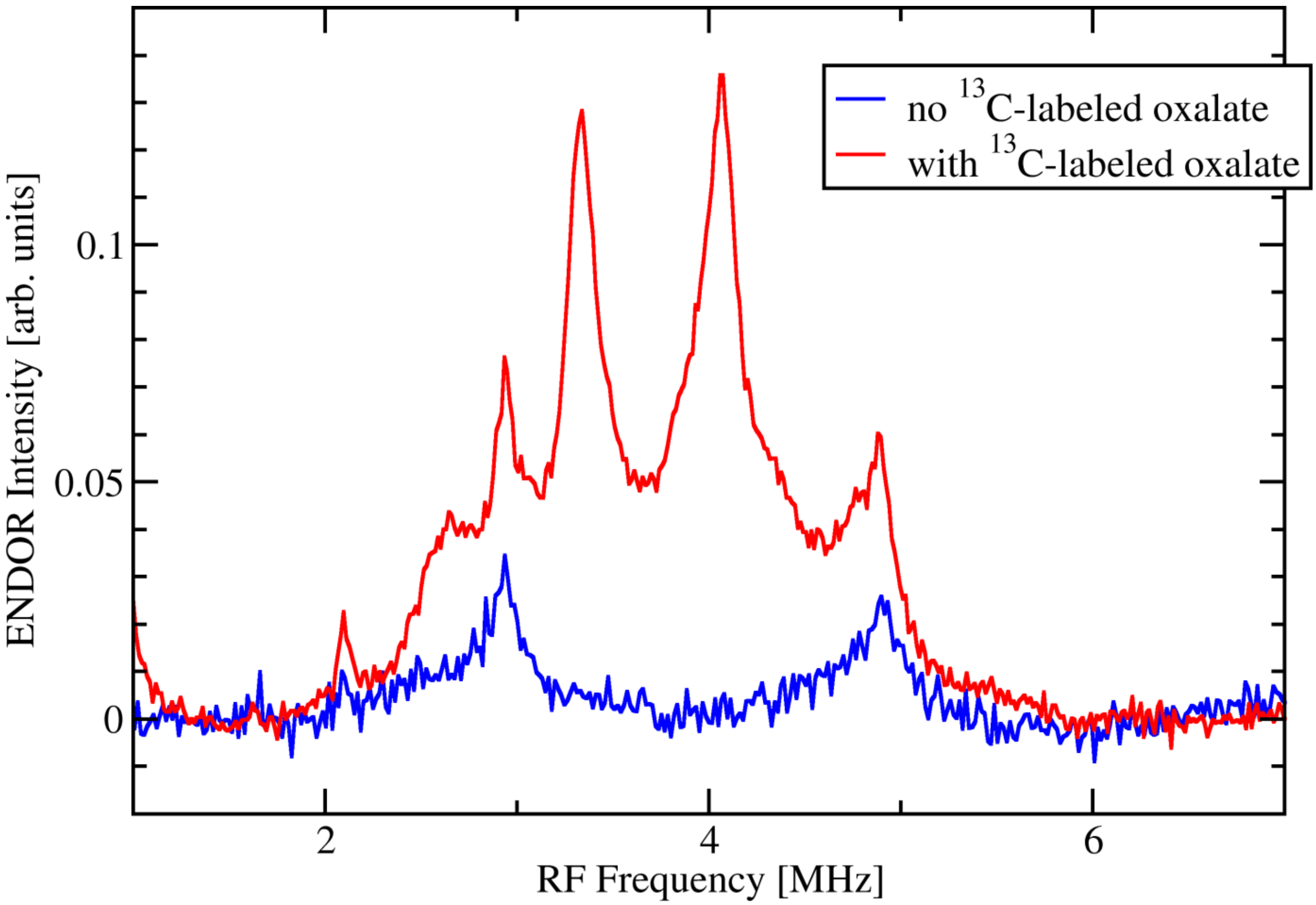

Figure S2: ENDOR spectra taken on the Mn(II) signal of W96F at pH 5.0 (blue) without ^{13}C-labeled oxalate and (red) with ^{13}C-labeled oxalate. The ENDOR spectra were baseline-corrected. The peaks near 3 and 5 MHz correspond to the 5th and 3rd harmonics of the proton ENDOR signals to allow for an internal reference at low RF frequencies. Both spectra are approximately normalized to these two signals. These spectra illustrate that oxalate binds to the active-site Mn(II) ion since the corresponding ^{13}C-ENDOR signals only show up in the presence of ^{13}C-labeled oxalate.

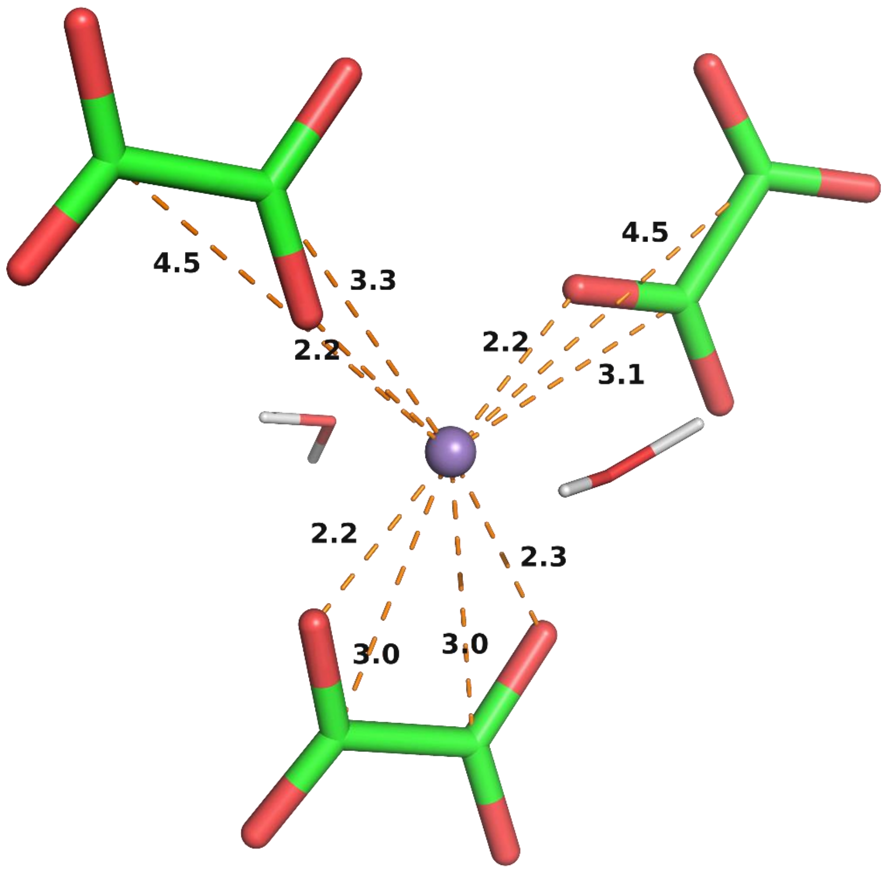

Figure S3a: Structure of $MnC_2O_4 \cdot 2H_2O$ (compound I in Lethbridge et al.) [1].

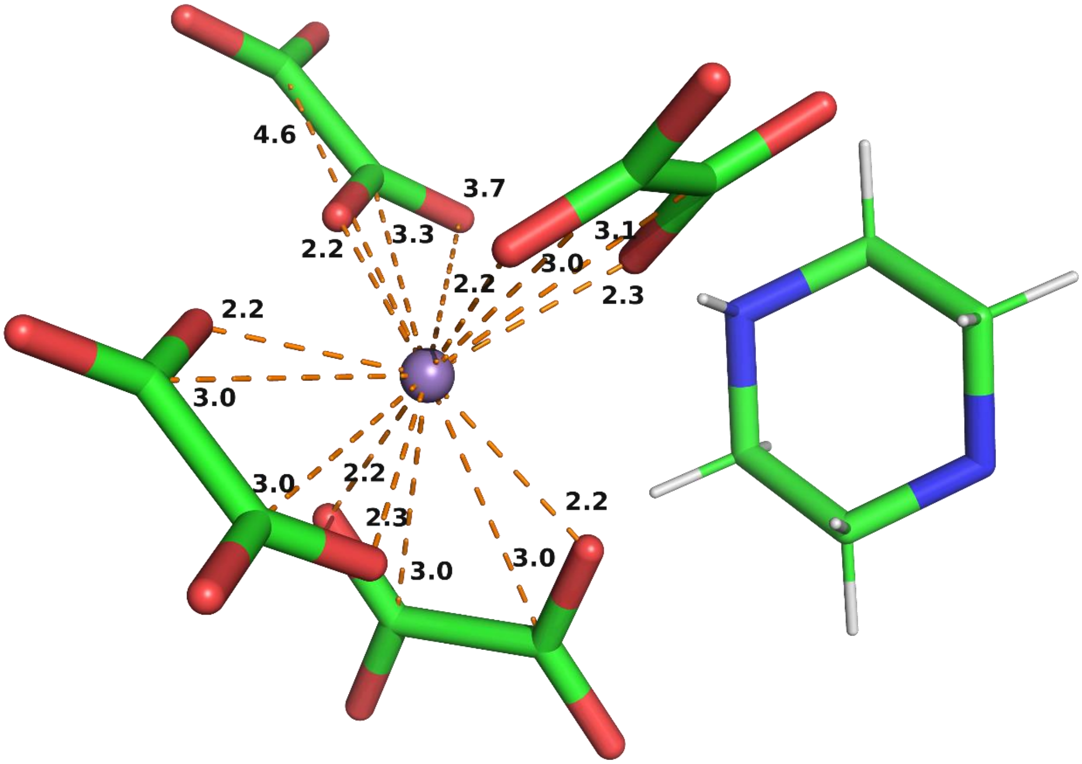

Figure S3b: Structure of [$C_4H_8(NH_2)_2$][$Mn_2(C_2O_4)_3$] (compound II in Lethbridge et al.) [1].

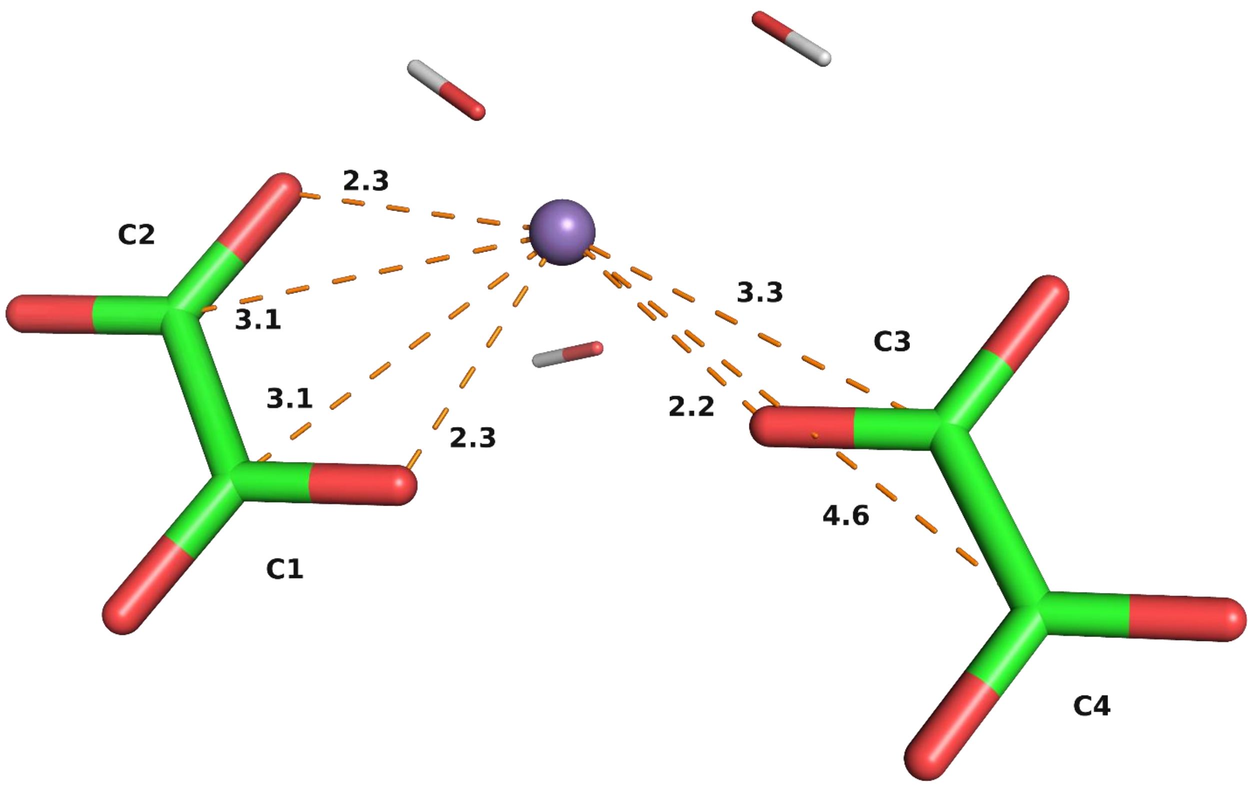

Figure S3c: Structure of $Mn_2(C_2O_4)(OH)_2$ (compound III in Lethbridge et al.) [1].

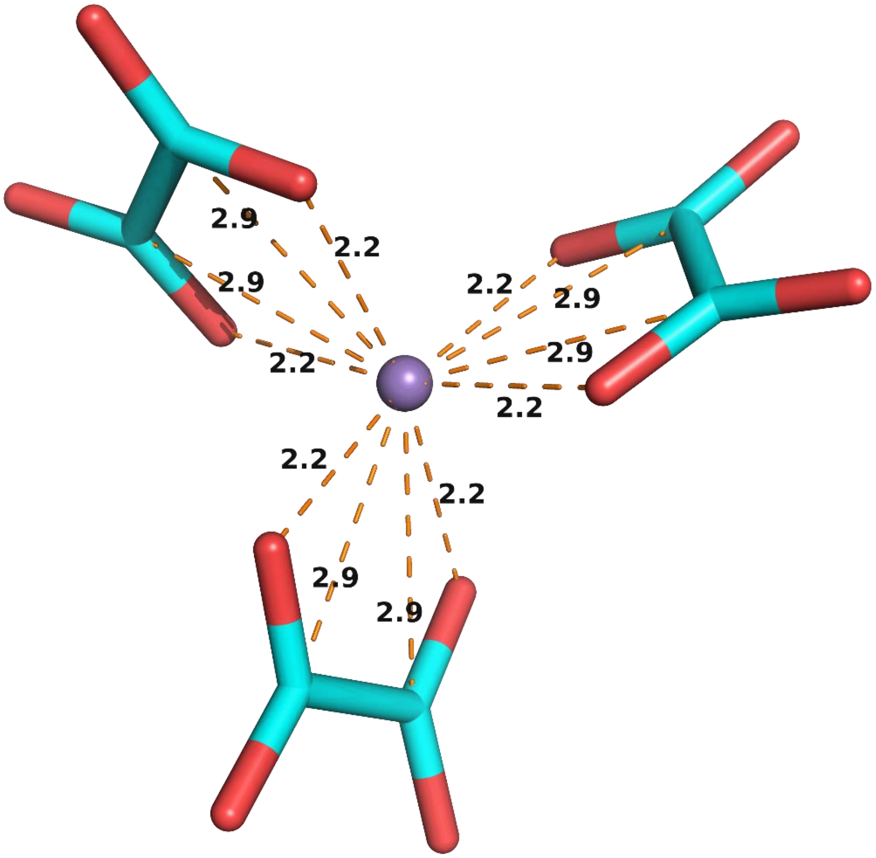

Figure S3d: Mn(II) coordinated by three oxalate dianions in a 3D $[Mn_2(C_2O_4)_3]_n^{2n-}$ anionic network by Habjanič et al. [2].

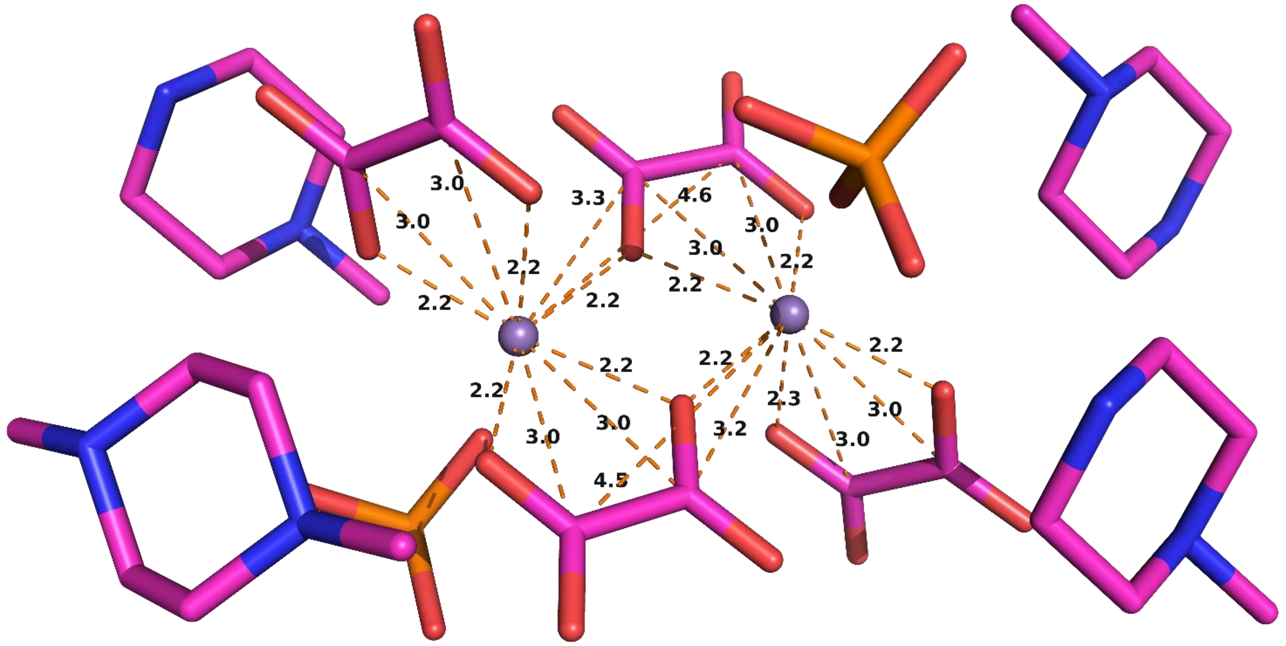

Figure S3e: Structure of $(H_2mpip)_{1.5}{\cdot}Mn_3(HPO_4)(H_2PO_4)(ox)_3$ (compound I in Luan et al.) [3]. mpip = 1-methylpiperazine, ox = oxalate.

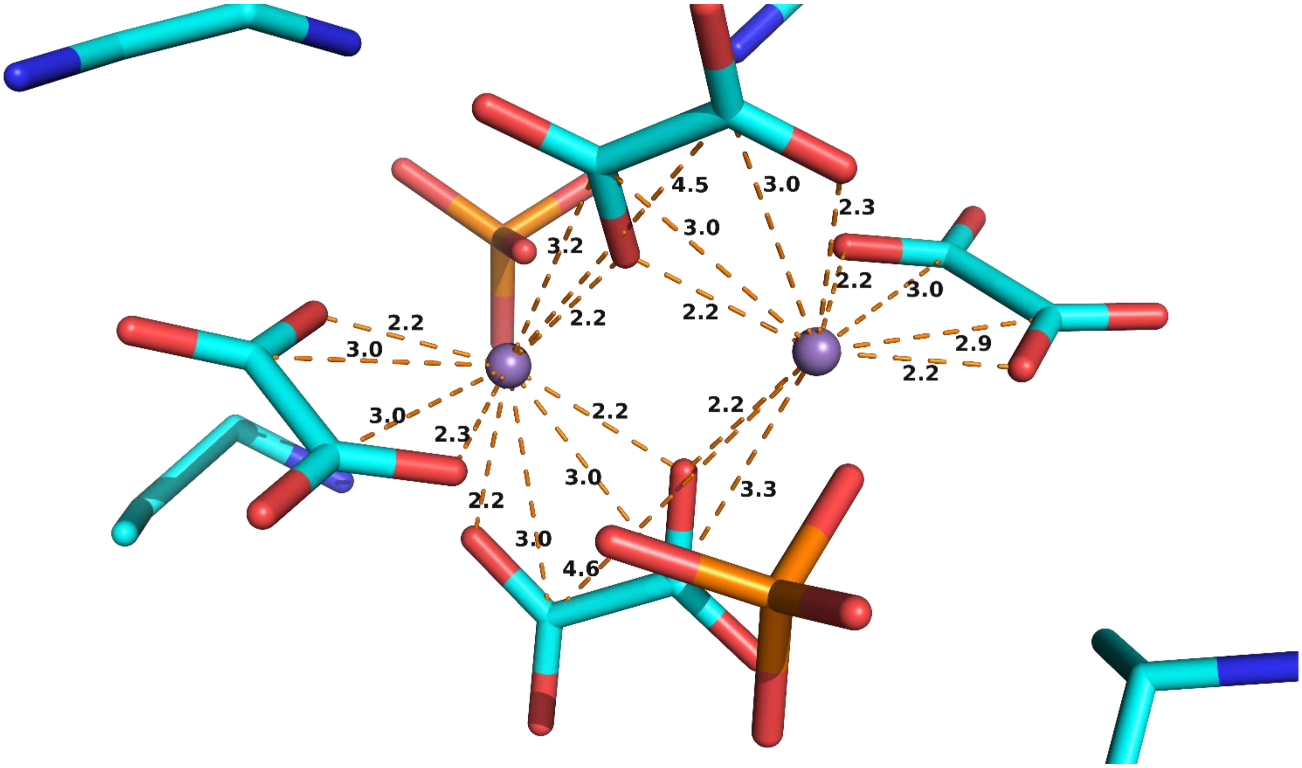

Figure S3f: Structure of $H_2dap{\cdot}Mn_2(HPO_4)(ox)_2$ *(compound II in Luan et al.) [3]. dap = 1,2-diaminopropane, ox = oxalate.*

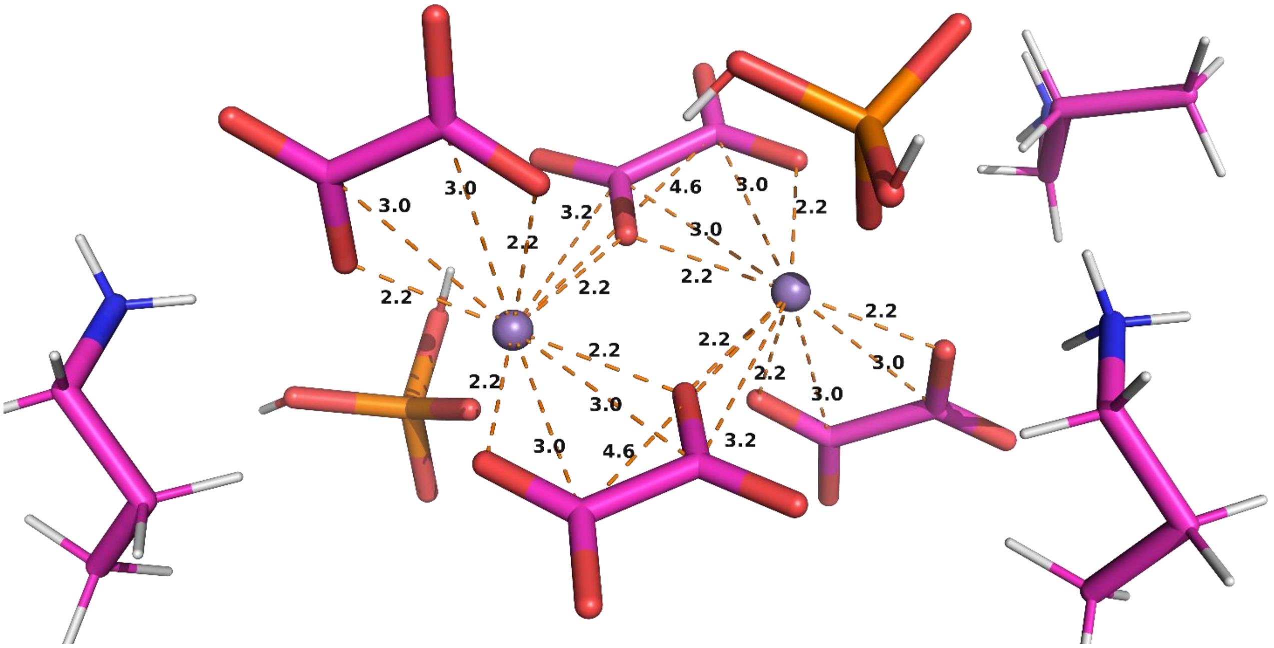

Figure S3g: Structure of Hpa·Mn2(H2PO4)(ox)2$_2$ *(compound III in Luan et al.) [3]. pa = propylamine, ox = oxalate.*

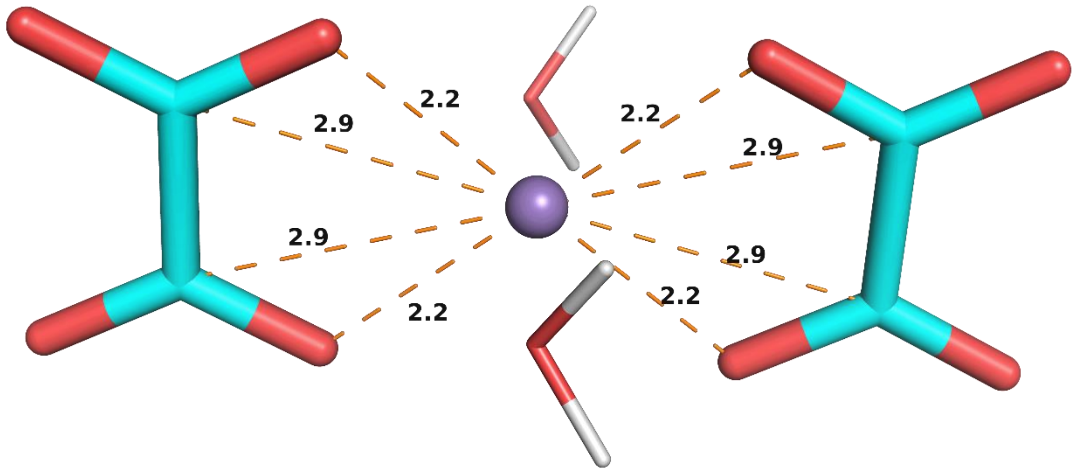

Figure S3h: Structure of $MnC_2O_4{\cdot}2H_2O$ (compound I in Puzan et al.) [4].

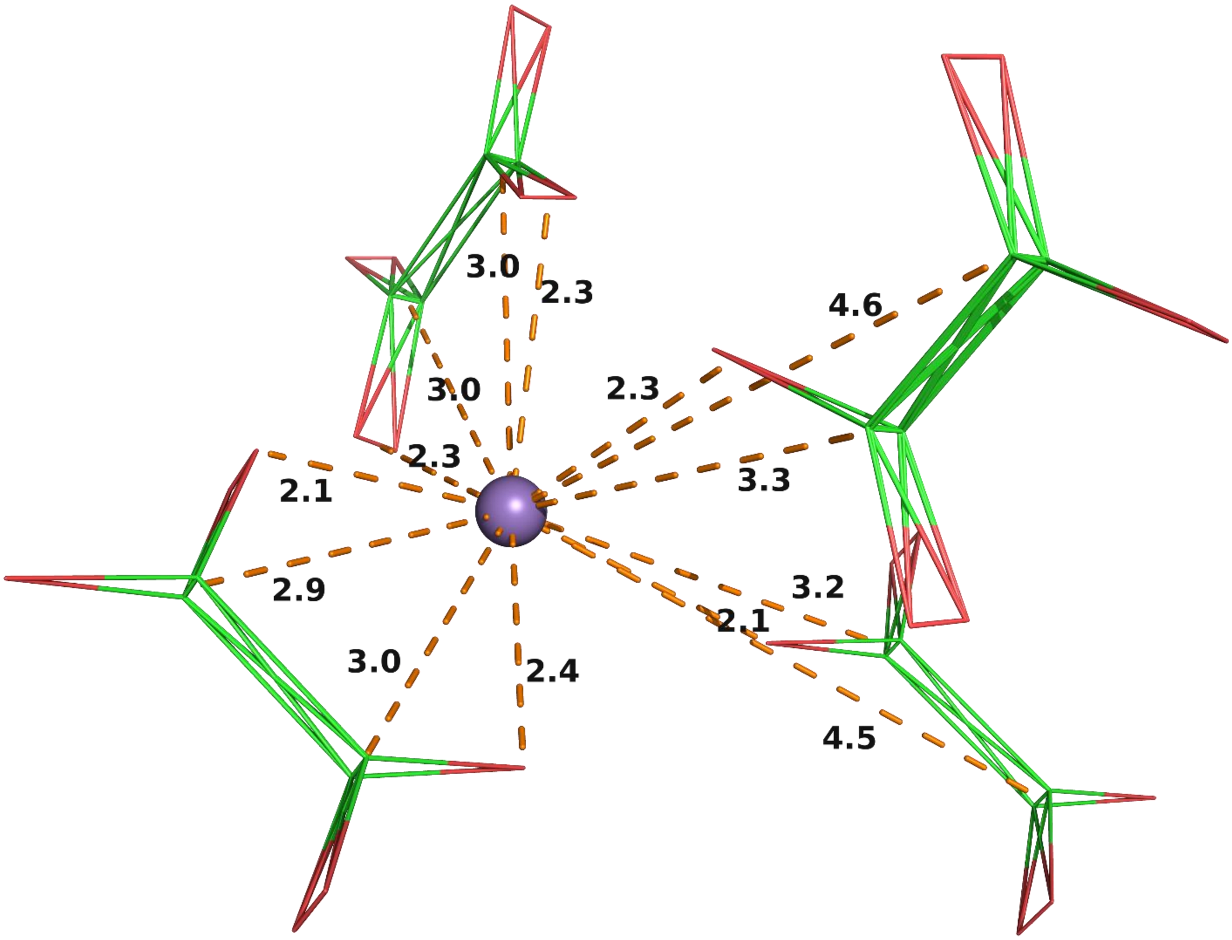

Figure S3i: Structure of anhydrous γ-MnC_2O_4 (compound II in Puzan et al.) [4].

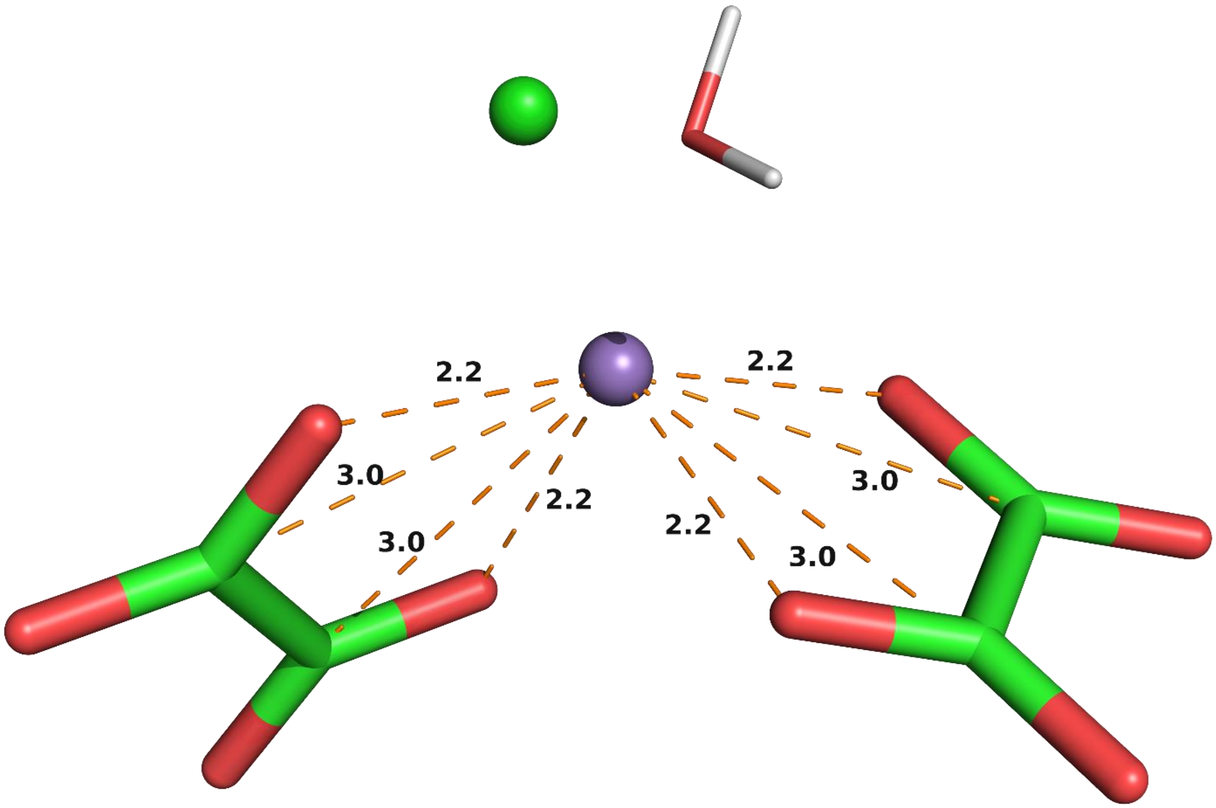

Figure S3j: Structure of the complex anion $MnCl(C_2O_4)(H_2O)]^-$ in $\{(CH_6N_3)[Mn(C_2O_4)Cl(H_2O)]H_2O\}n$ (compound I in Sehimi et al.) [5].

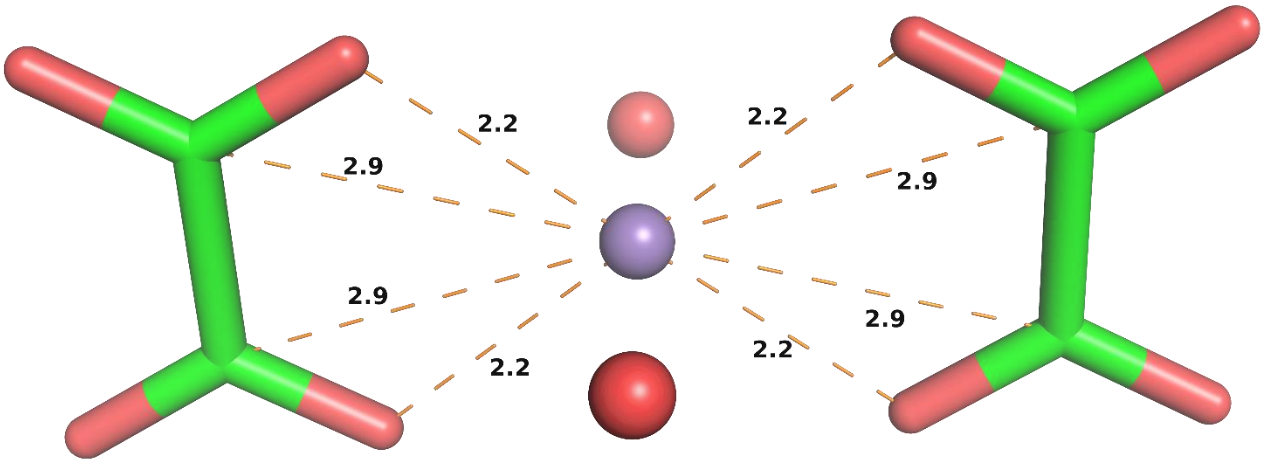

Figure S3k: Structure of Mn(II) oxalate dihydrate from Deyrieux et al. [6].

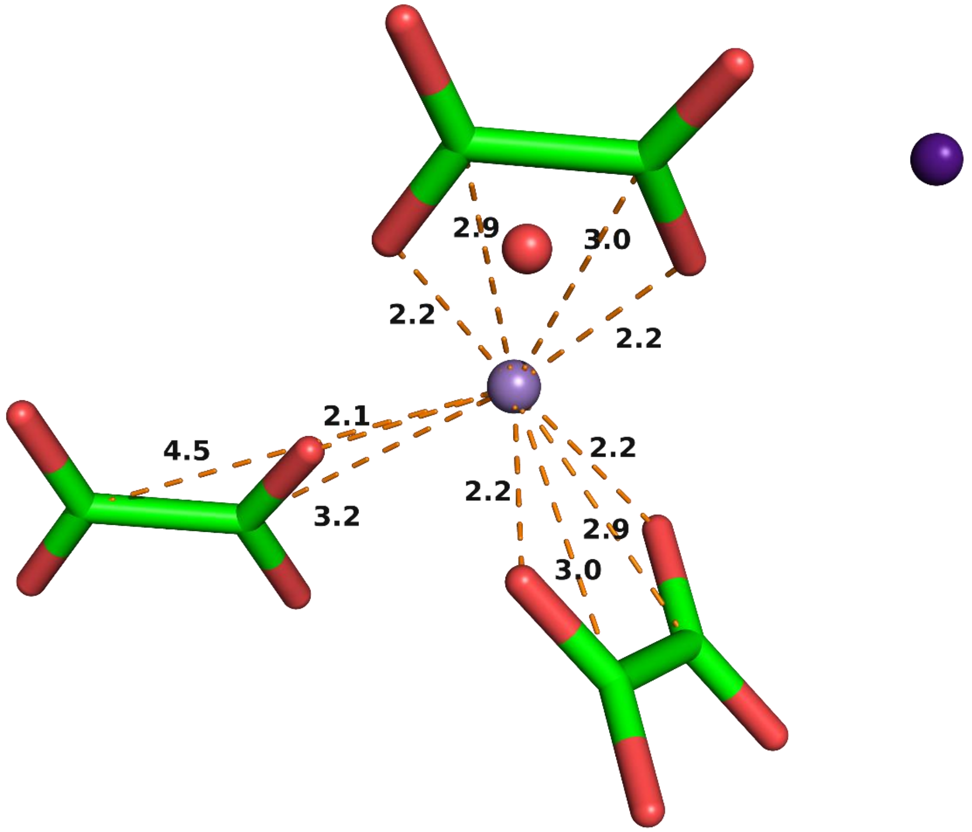

Figure S3l: Structure of catena(Cesium aqua-tris(μ_2-oxalato)-di-manganese dihydrate) from Siems and Löhn [7].

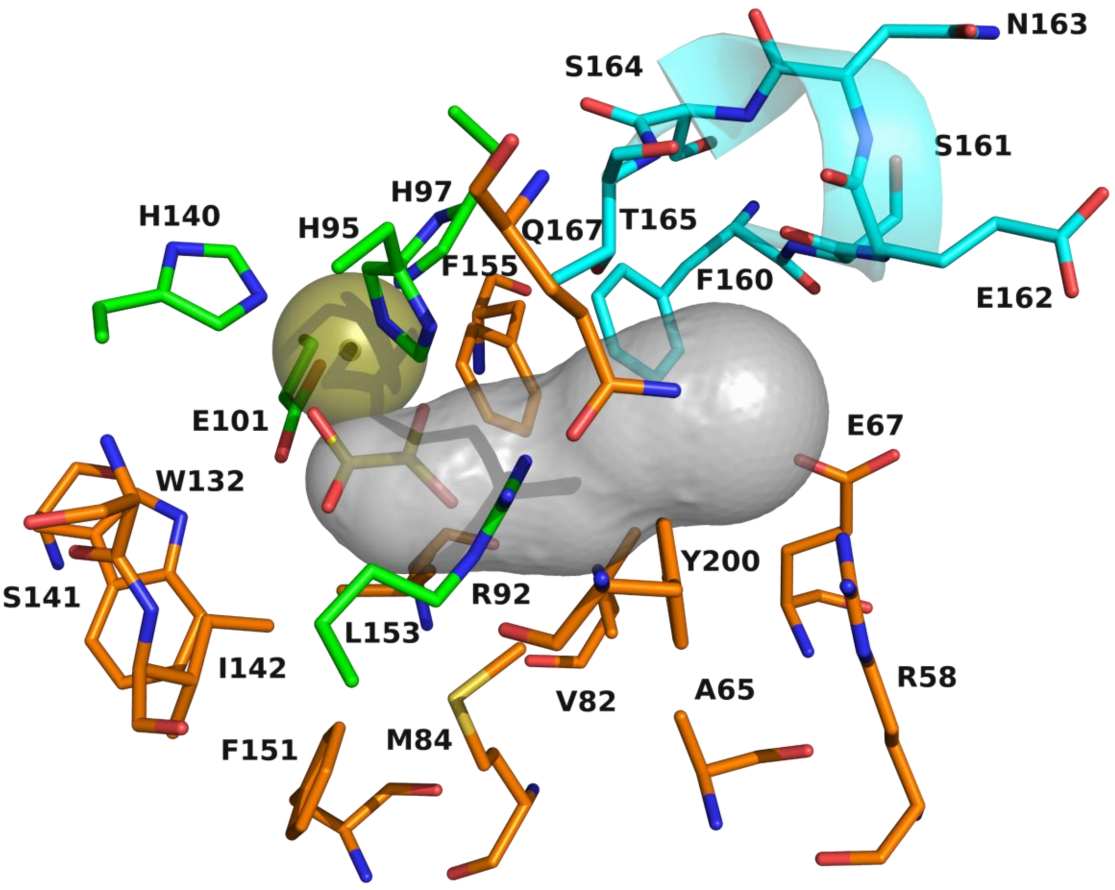

Figure S4a: Result of a Caver 3.0 calculation [8] indicating the solvent channel (transparent grey volume) to the active site Mn (olive ball with shaded outline of its van der Waals radius). The bi-dentate binding pose of the substrate oxalate is indicated inside the channel. Green residues are the ligands binding the N-terminal Mn ion plus residue R92 in the second coordination shell which makes hydrogen bonds to the substrate. The flexible SENST161–165 loop is shown in its open position and indicated in cyan color. Other residues surrounding the channel region are indicated in orange color.

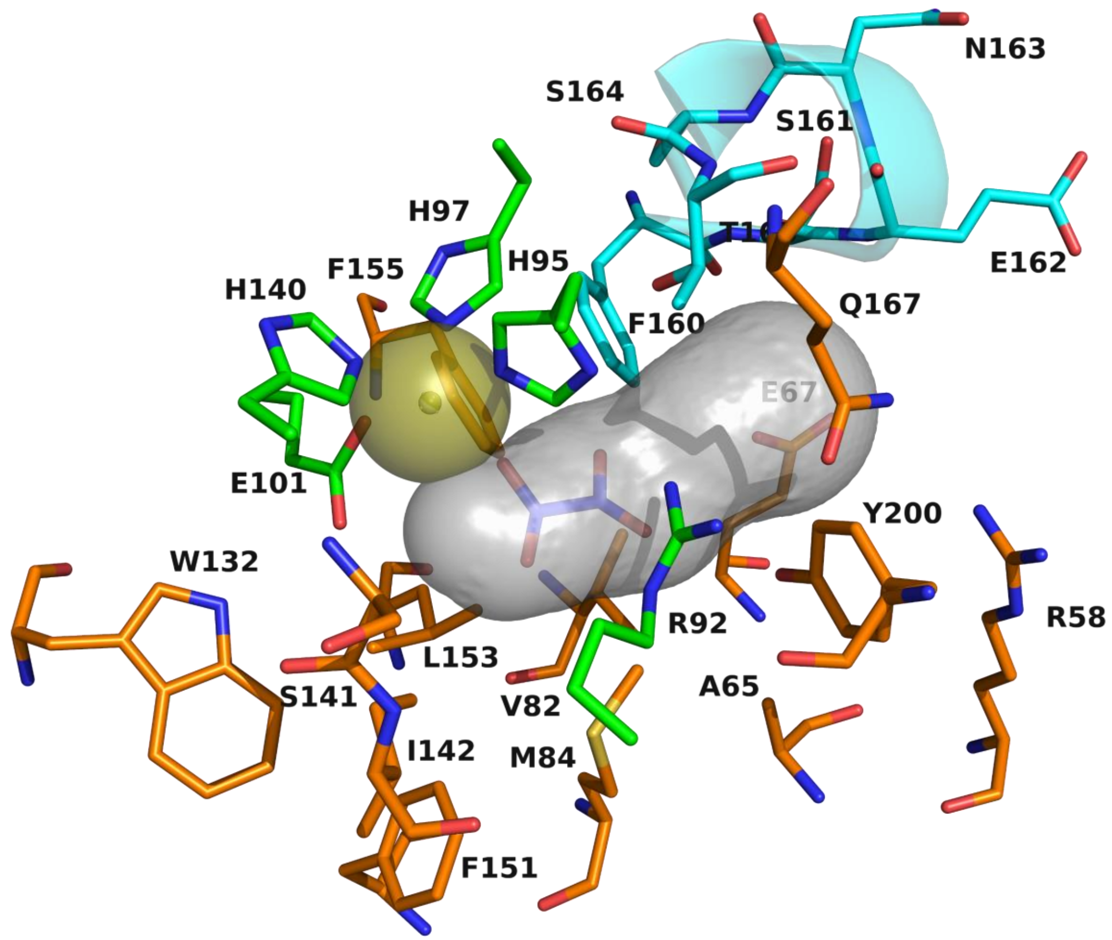

Figure S4b: Result of a Caver 3.0 calculation [8] indicating the solvent channel (transparent grey volume) to the active site Mn (olive ball with shaded outline of its van der Waals radius). The mono-dentate binding pose of the substrate oxalate is indicated inside the channel. Green residues are the ligands binding the N-terminal Mn ion plus residue R92 in the second coordination shell which makes hydrogen bonds to the substrate. The flexible SENST161–165 loop is shown in its open position and indicated in cyan color. Other residues surrounding the channel region are indicated in orange color.

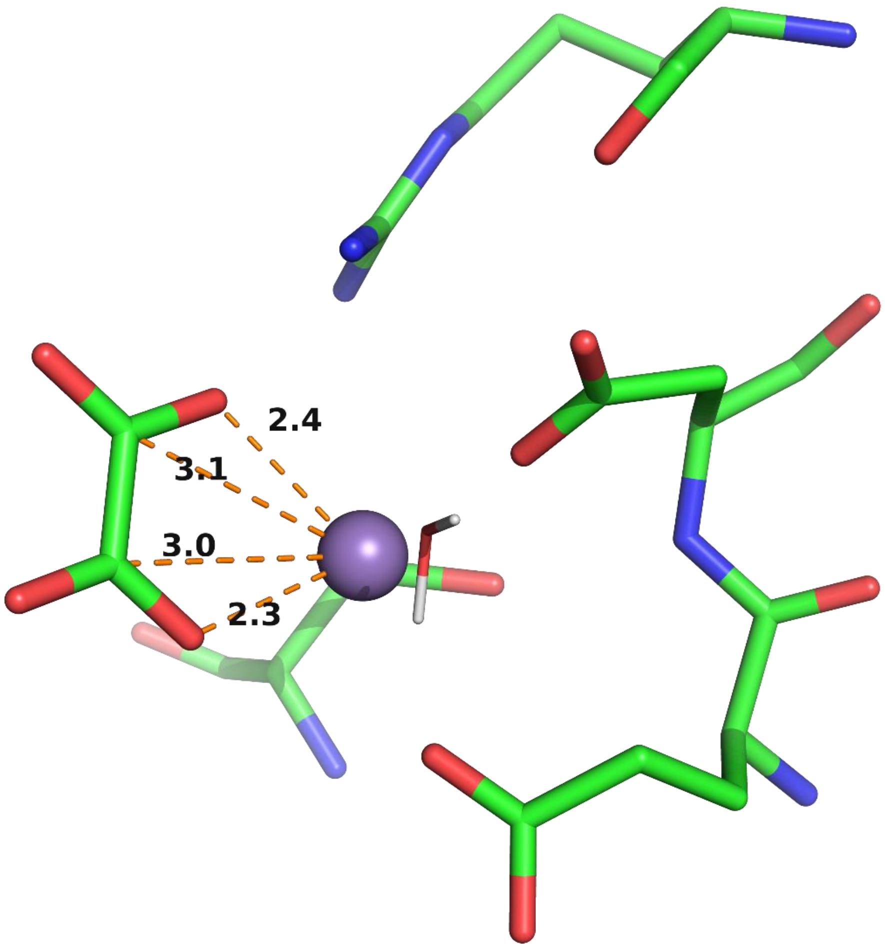

Figure S5a: Mn coordination of oxalate in Human Mitochondrial NAD(P)$^{+}$-Dependent Malic Enzyme (PDB #1DO8).

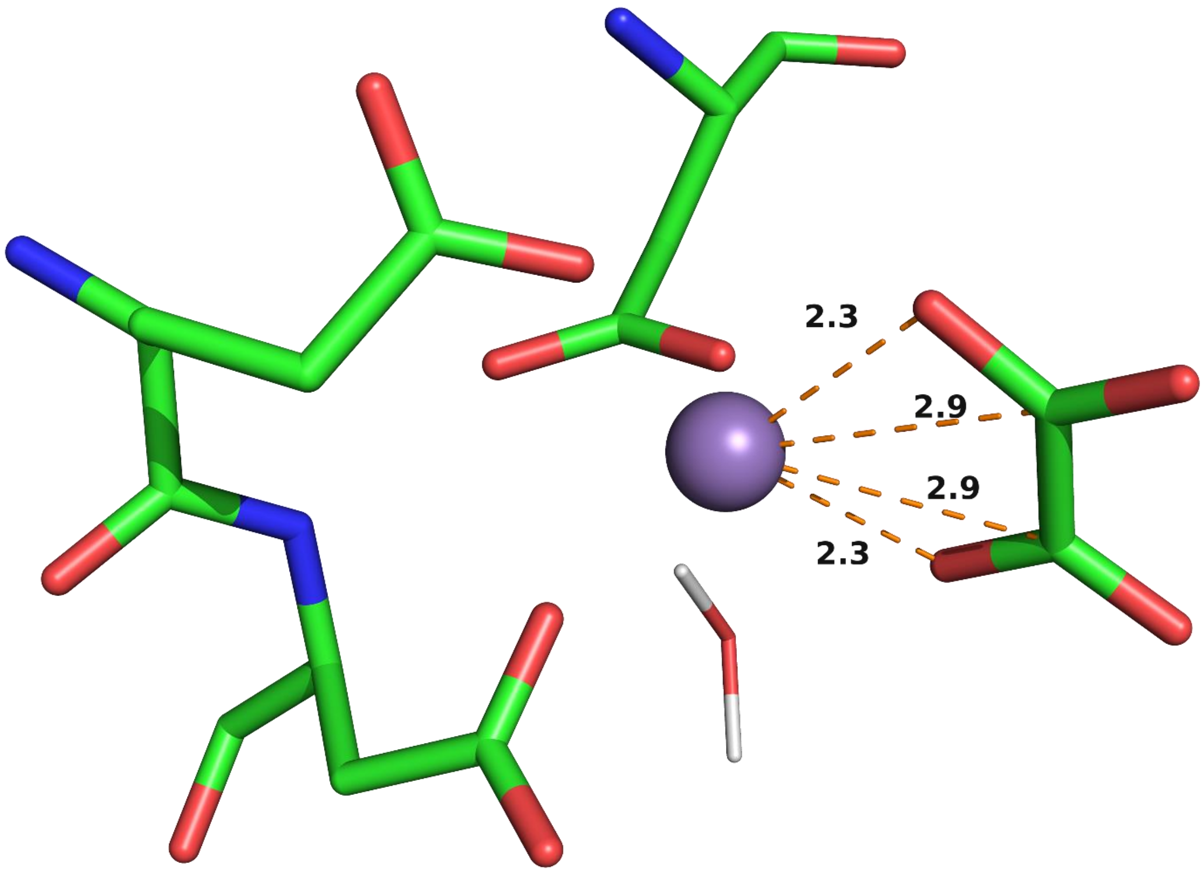

Figure S5b: Mn coordination of oxalate in Pigeon Liver Malic Enzyme (PDB #1GQ2).

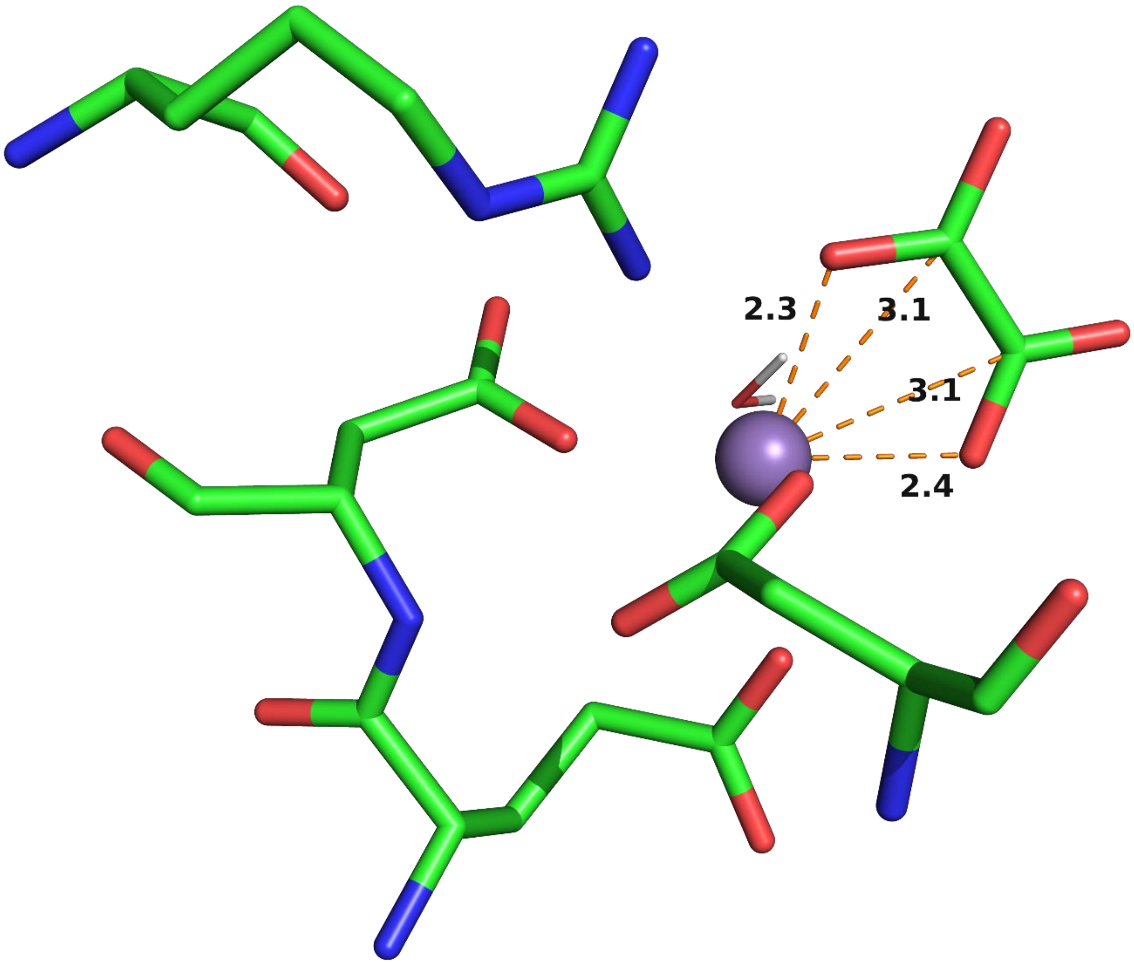

Figure S5c: Mn coordination of oxalate in Human Mitochondrial NAD(P)$^{+}$-Dependent Malic Enzyme (PDB #1GZ3).

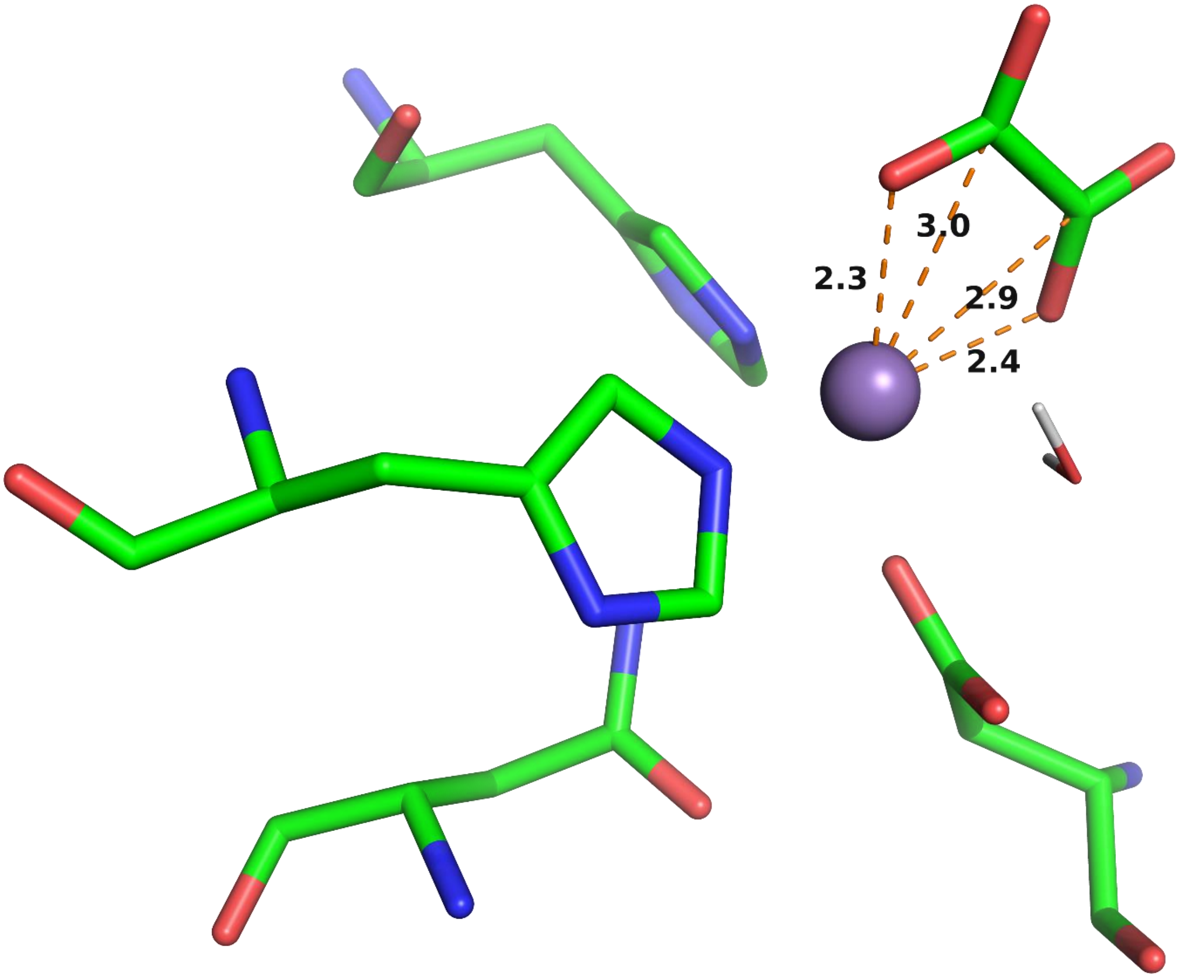

Figure S5d: Mn coordination of oxalate in 4-hydroxy-2-ketovalerate aldolase (DmpG)/acylating acetaldehyde dehydrogenase (DmpF), (PDB #1NVM)

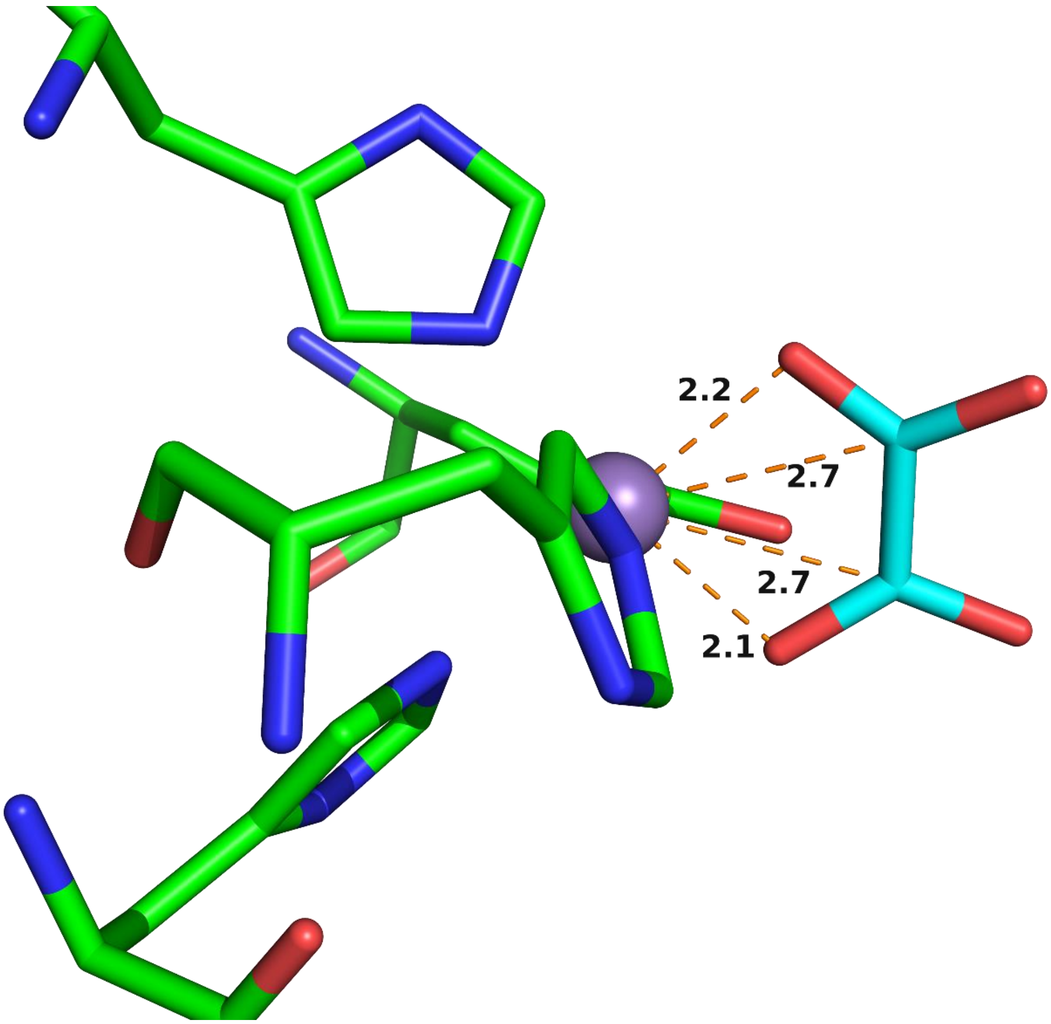

Figure S5e: Mn coordination of oxalate in Putative Oxalate Decarboxylase (TM1287) from Thermotoga maritima, (PDB #1O4T)

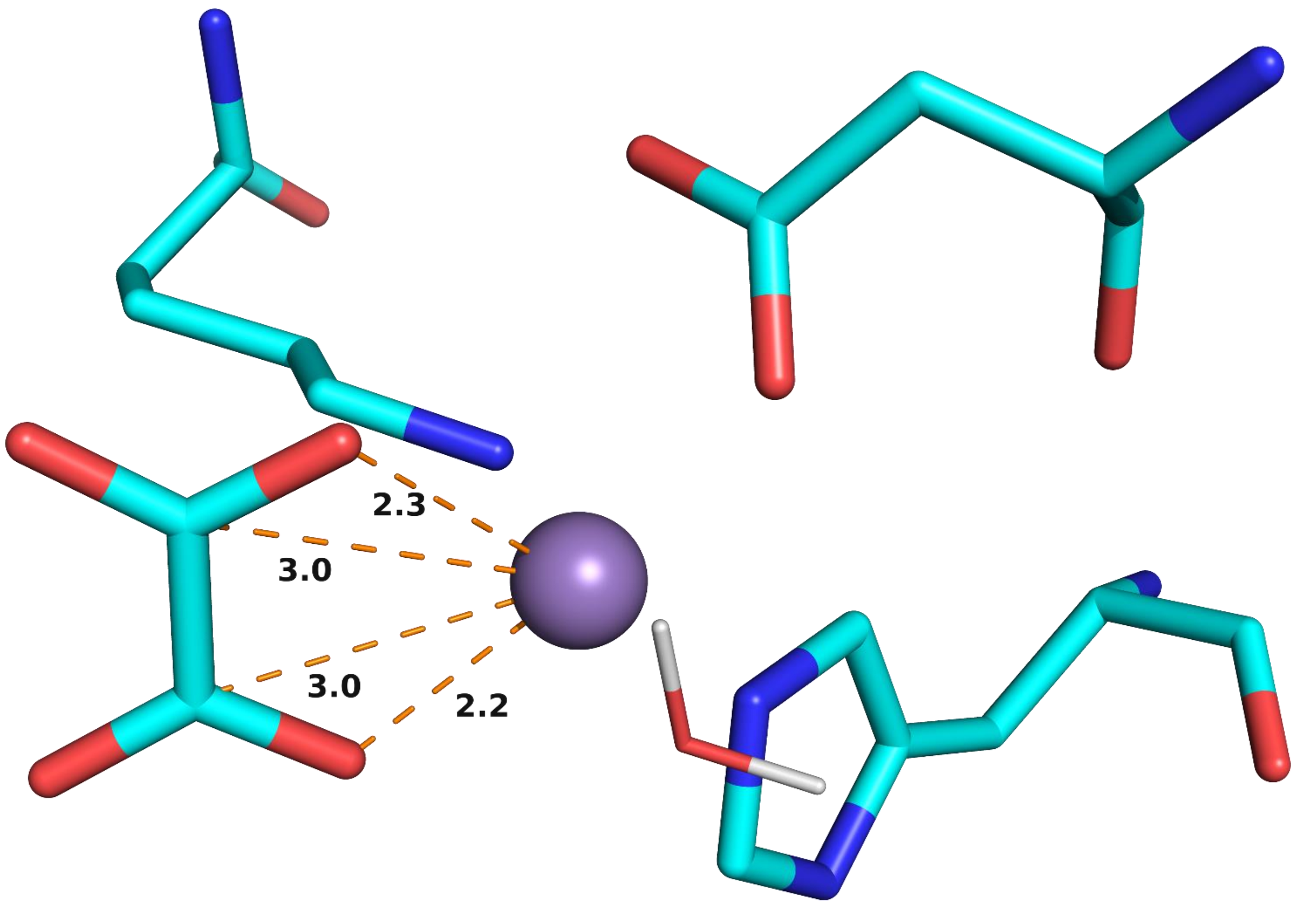

Figure S5f: Mn coordination of oxalate in Cytosolic phosphoenolpyruvate carboxykinase (cPEPCK), (PDB #2RK7).

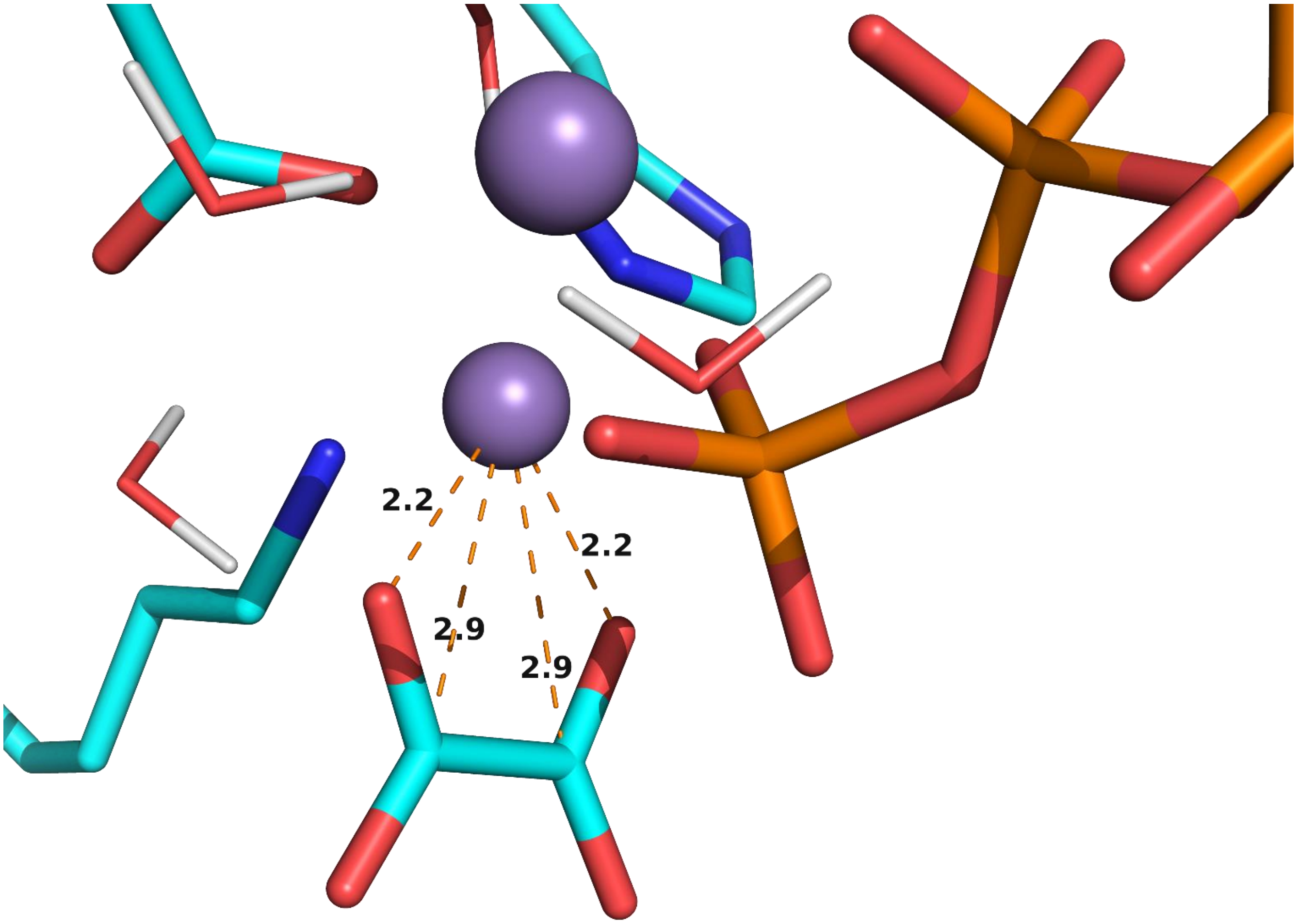

Figure S5g: Mn coordination of oxalate in Cytosolic phosphoenolpyruvate carboxykinase (cPEPCK), (PDB #3DT2).

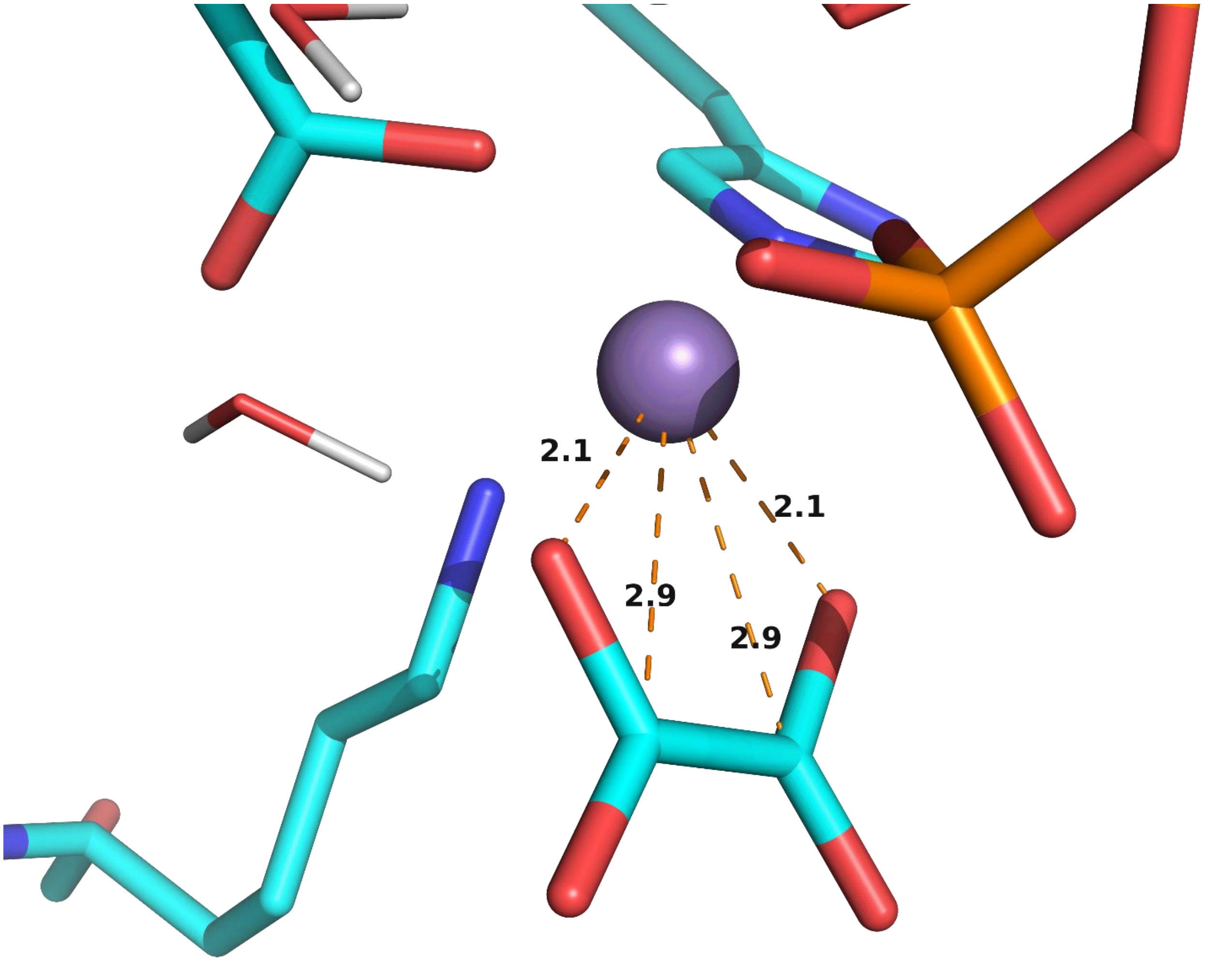

Figure S5h: Mn coordination of oxalate in Cytosolic phosphoenolpyruvate carboxykinase (cPEPCK), (PDB #3DT4).

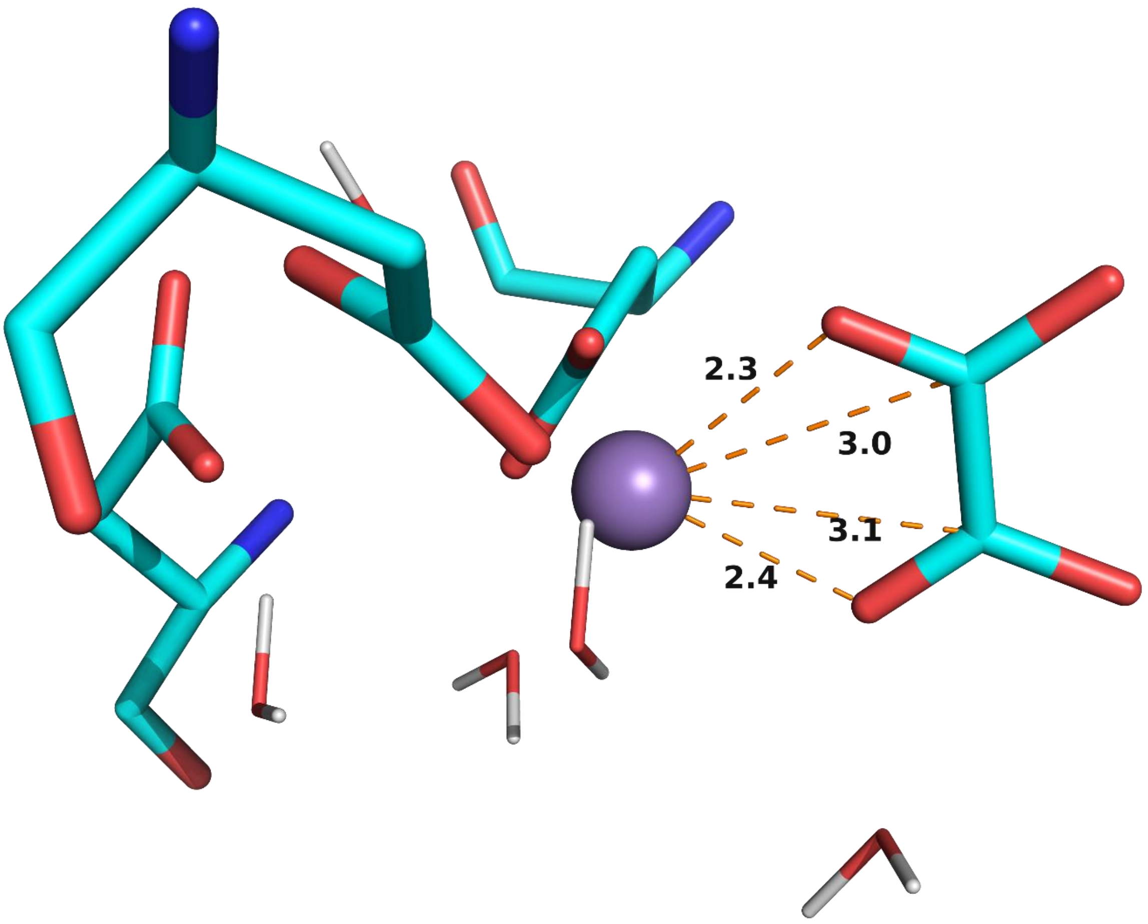

Figure S5i: Mn coordination of oxalate in Oxalacetate Acetylhydrolase, (PDB #3M0K).

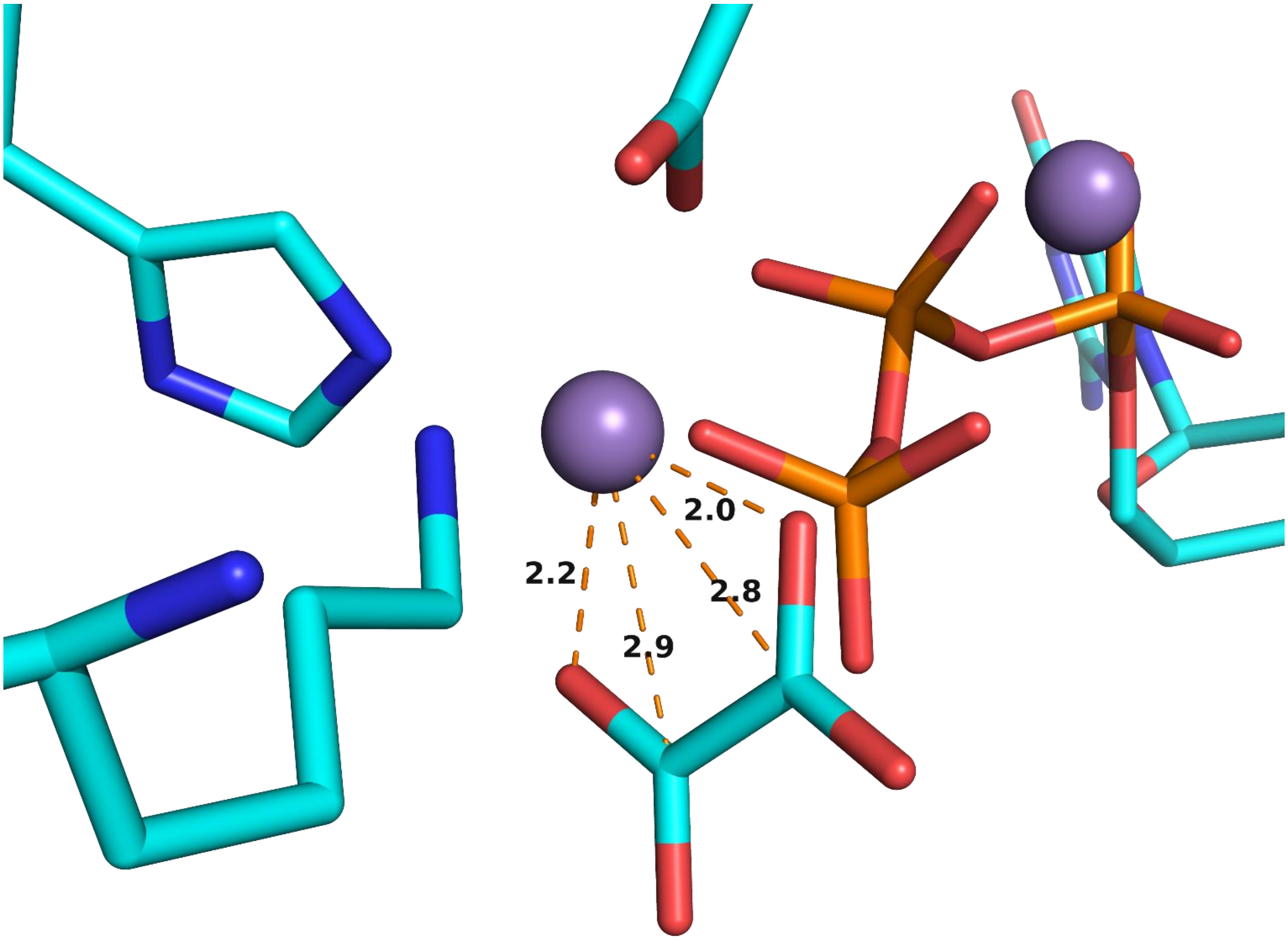

Figure S5j: Mn coordination of oxalate in Cytosolic phosphoenolpyruvate carboxykinase (cPEPCK) mutant A467G, (PDB #3MOF).

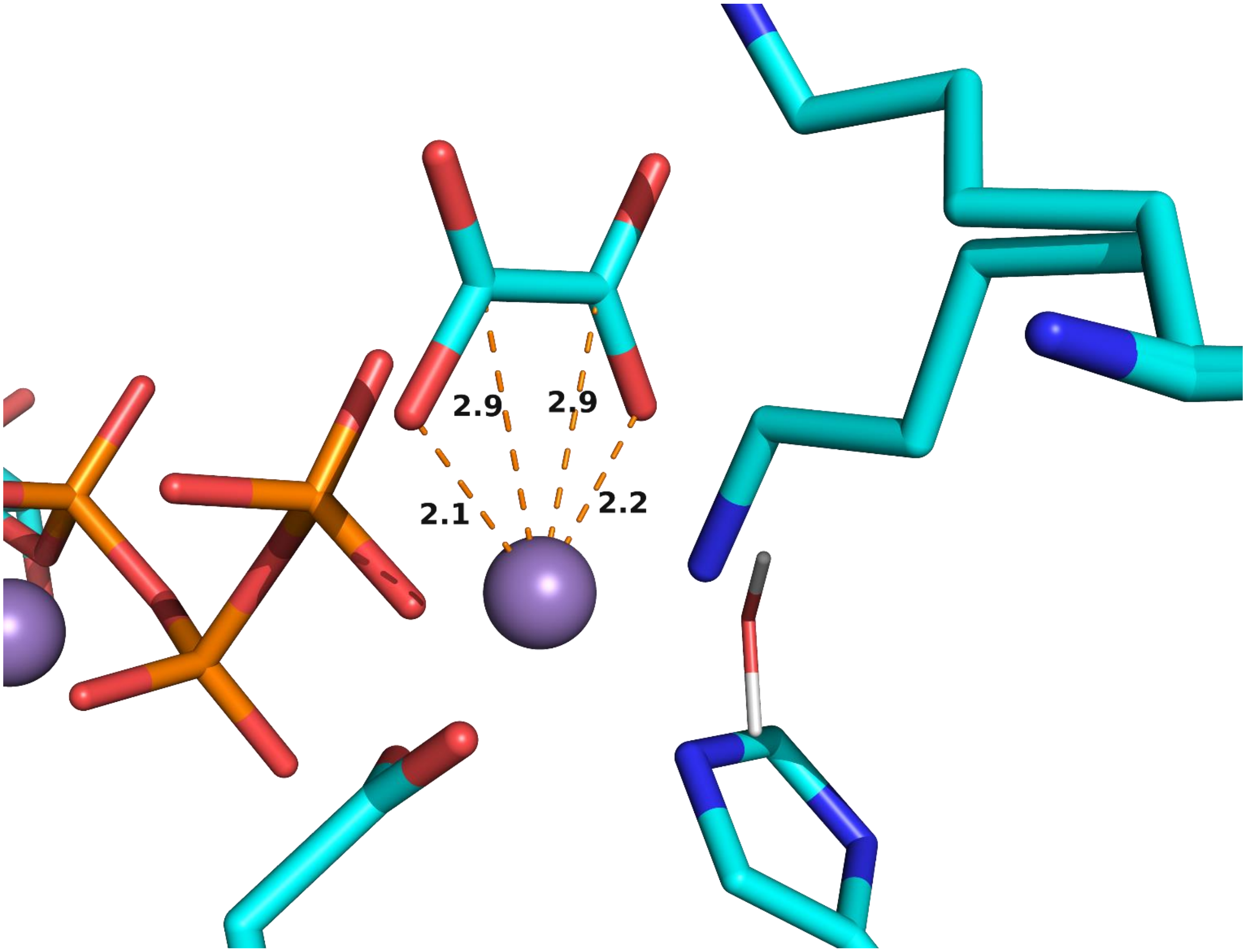

Figure S5k: Mn coordination of oxalate in Rat Cytosolic phosphoenolpyruvate carboxykinase (cPEPCK) Ld_1g, (PDB #4GMU).

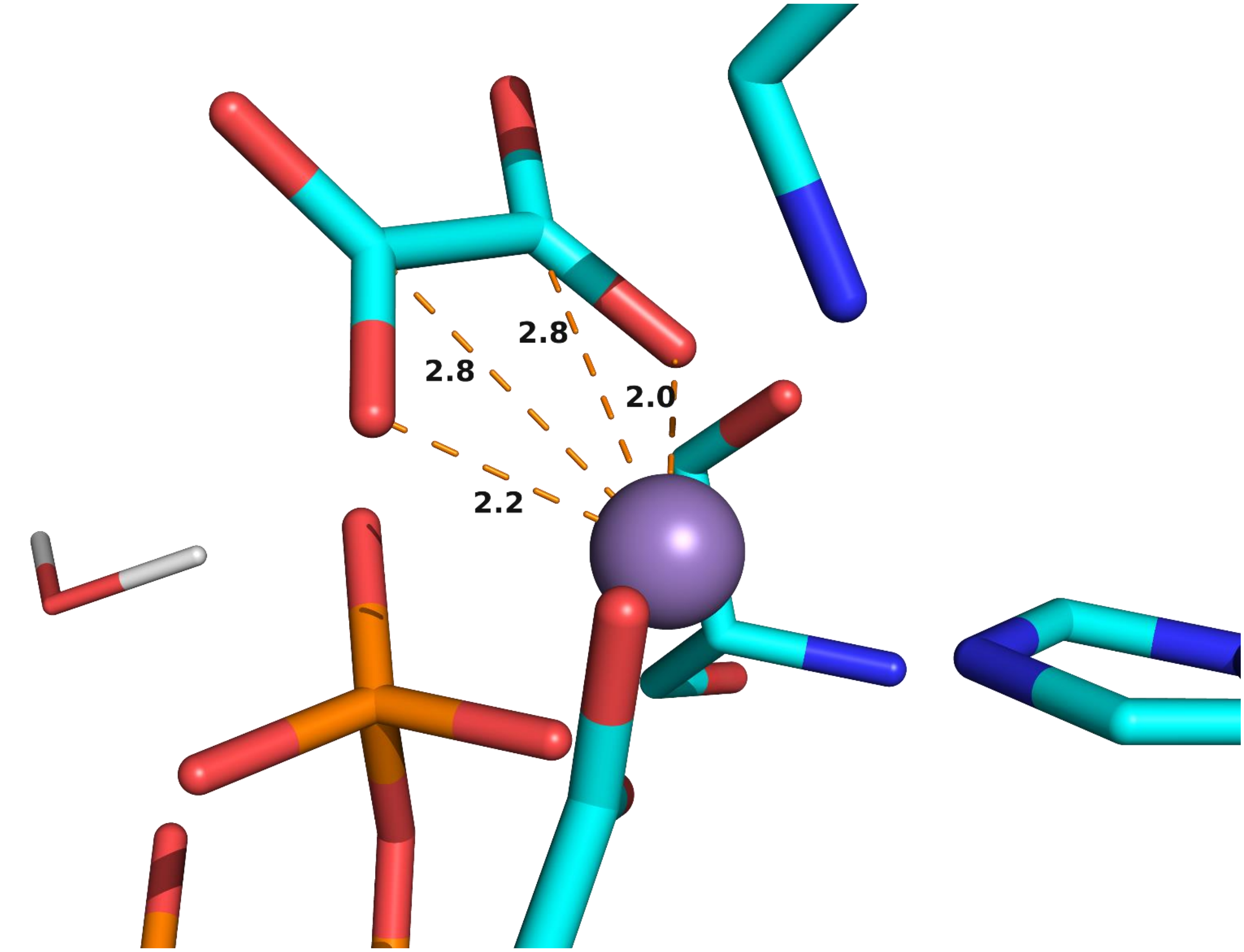

Figure S5l: Mn coordination of oxalate in Rat Cytosolic phosphoenolpyruvate carboxykinase (cPEPCK) Ld_2g, (PDB #4GNM).

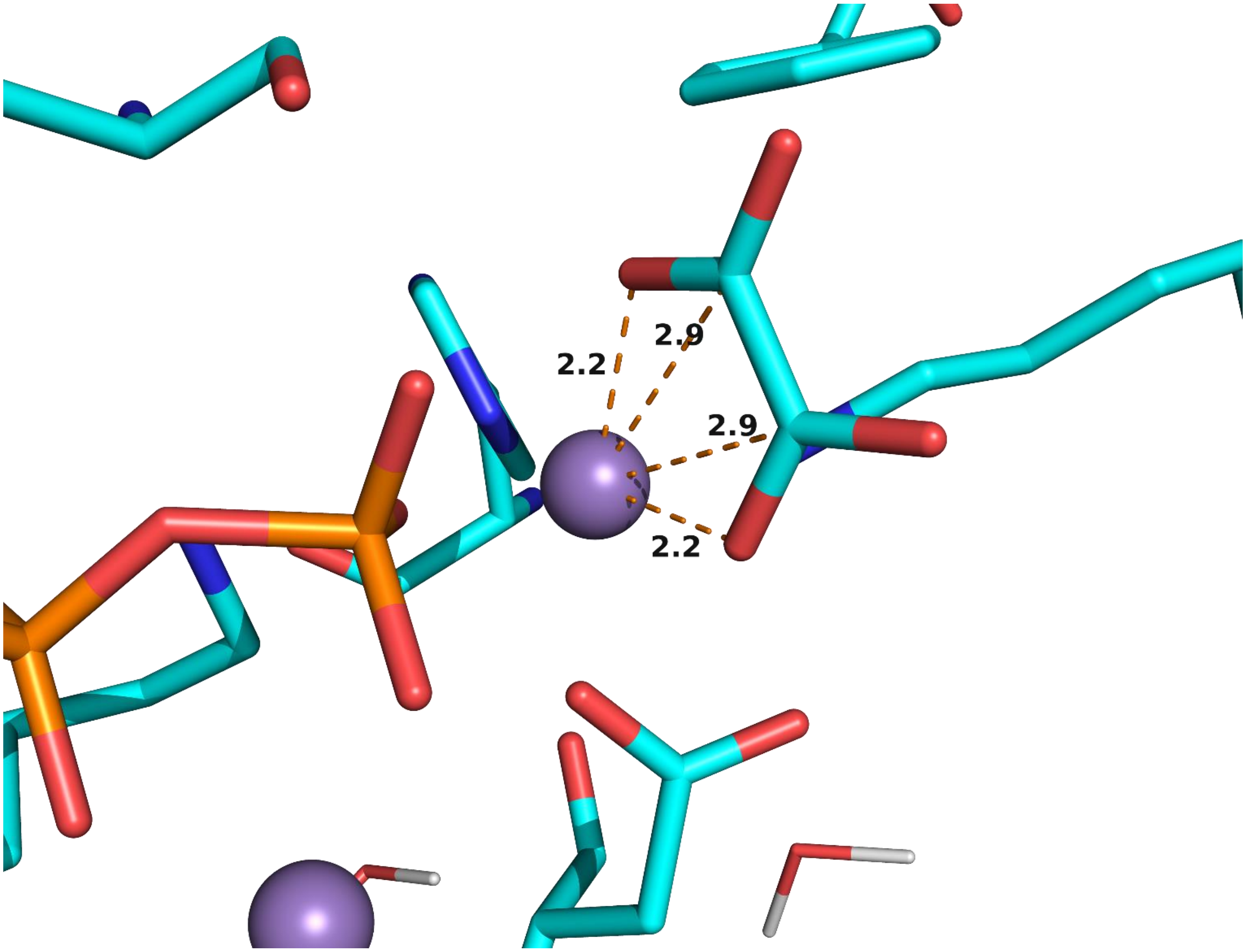

Figure S5m: Mn coordination of oxalate in Rat Cytosolic phosphoenolpyruvate carboxykinase (cPEPCK) Ld_3g, (PDB #4GNQ).

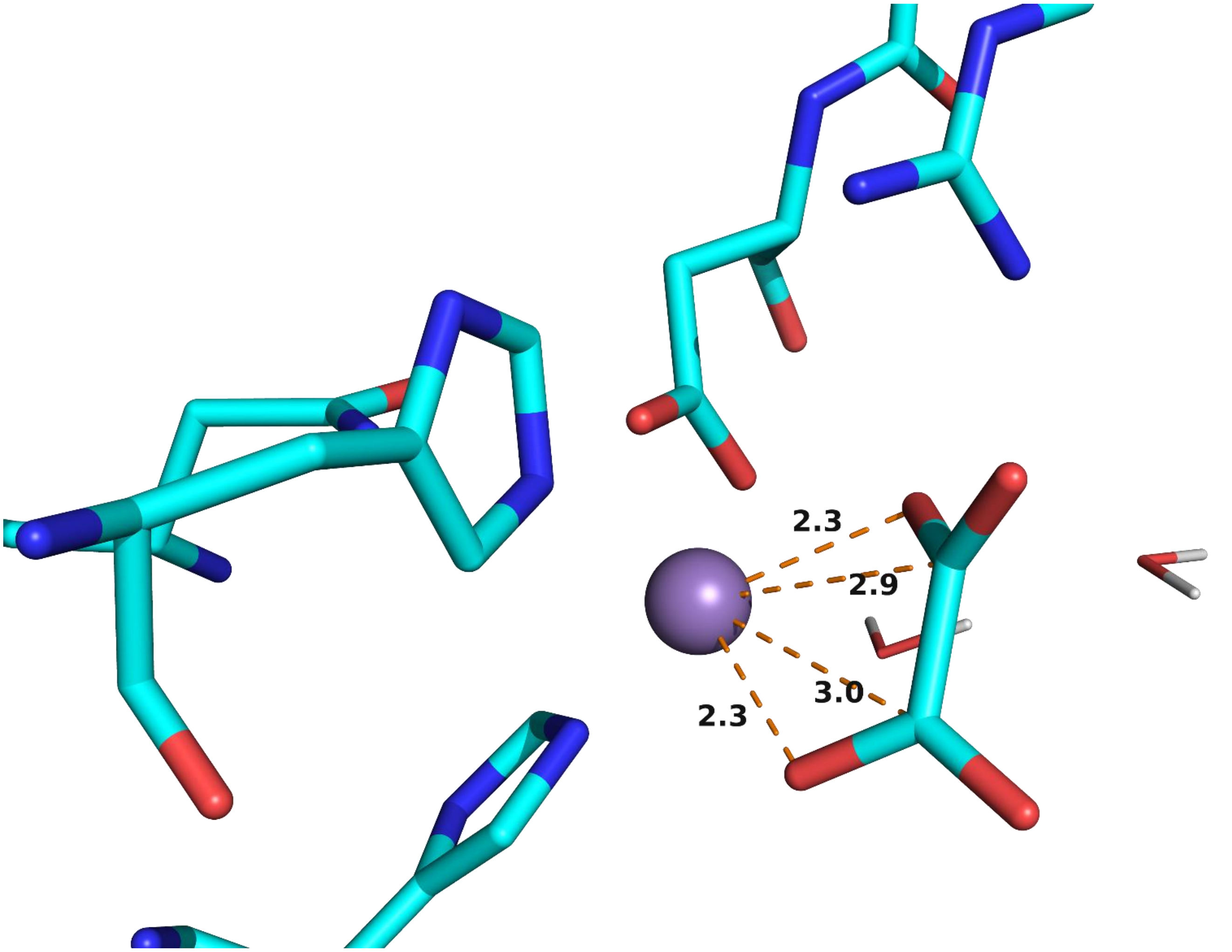

Figure S5n: Mn coordination of oxalate in Aldolase-Dehydrogenase Complex from the Cholesterol Degradation Pathway of Mycobacterium tuberculosis, (PDB #4JN6).

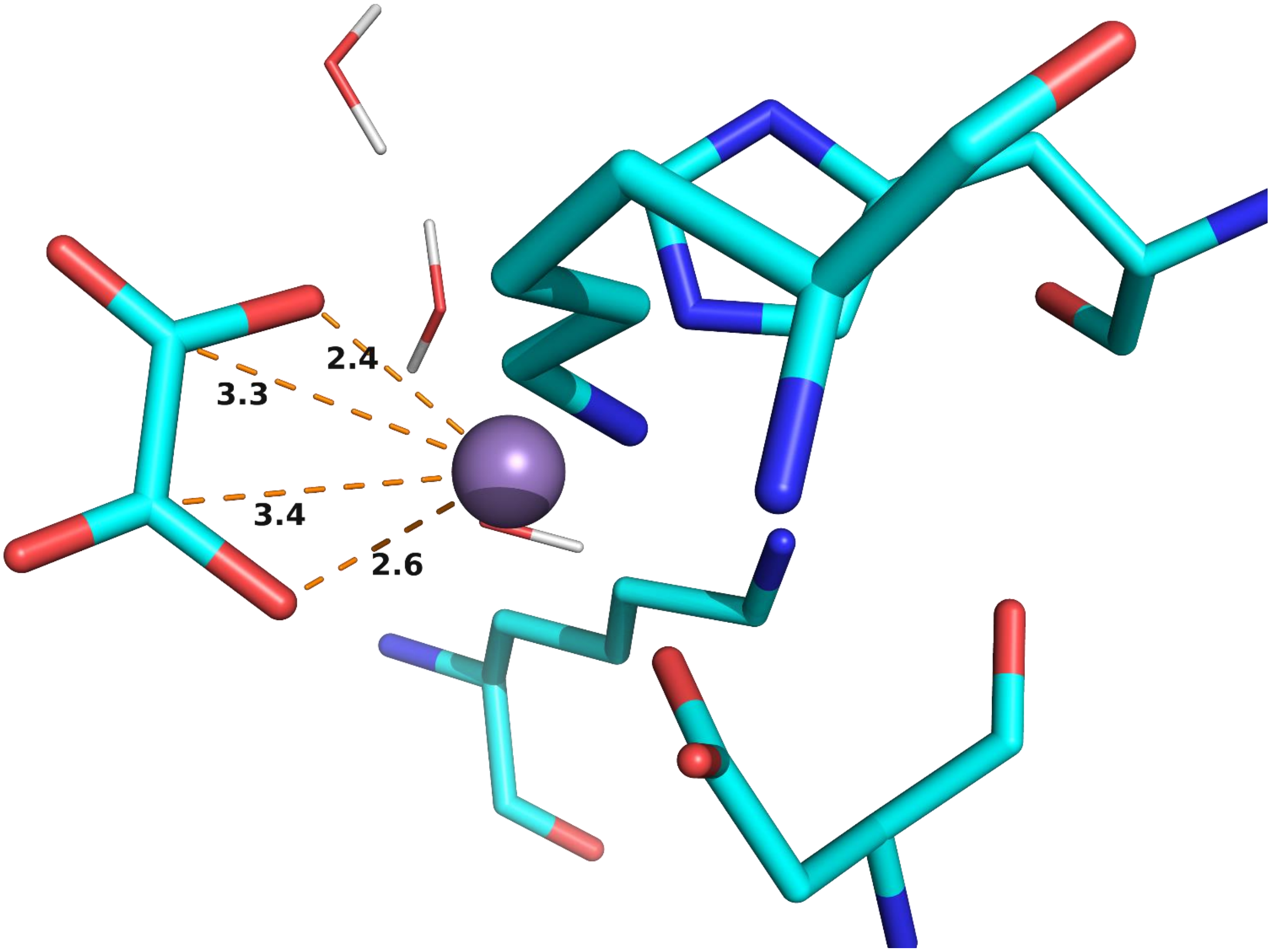

Figure S5o: Mn coordination of oxalate in PEPCK (Rv0211) from Mycobacterium tuberculosis, (PDB #4WIU).

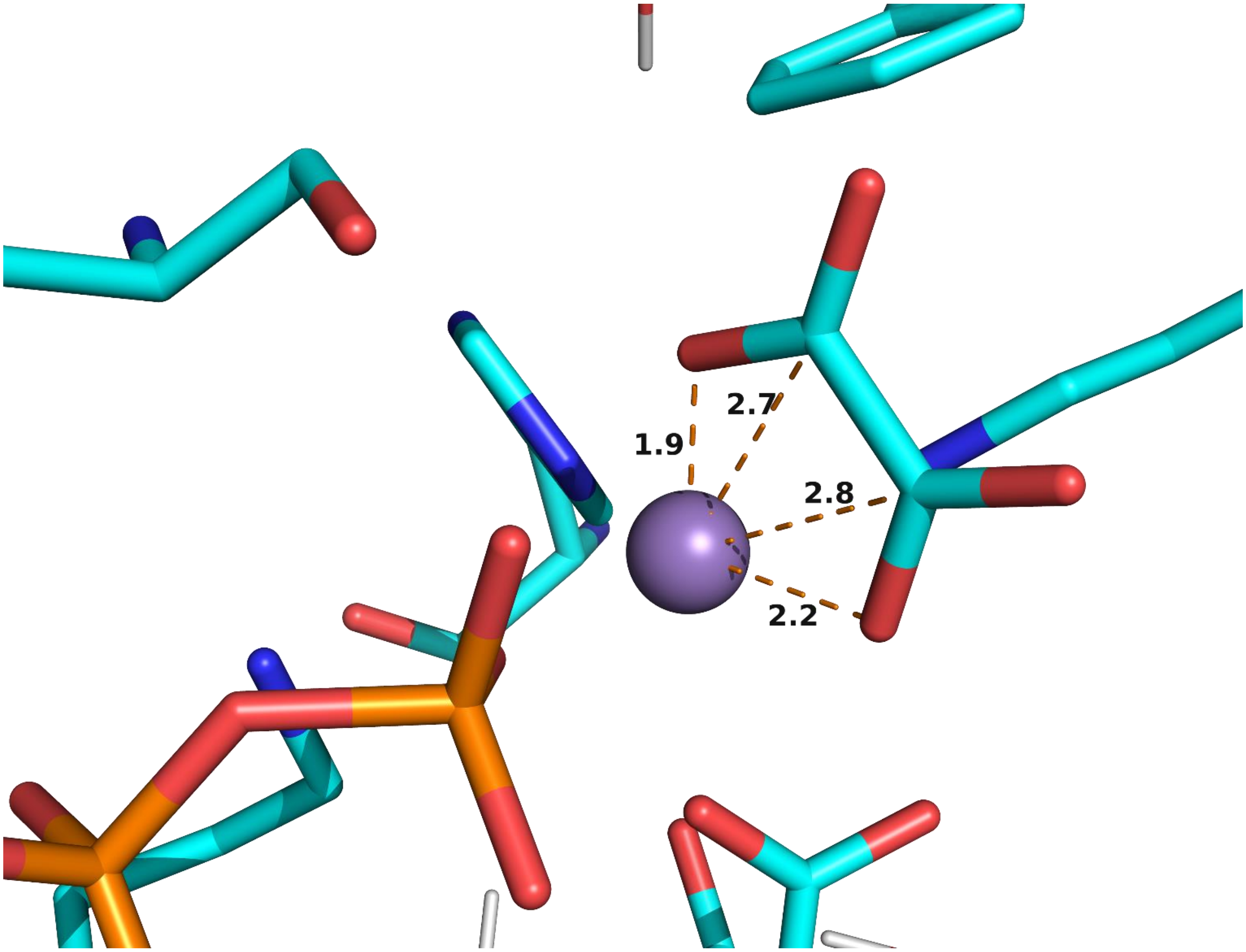

Figure S5p: Mn coordination of oxalate in Cytosolic PEPCK Variant E89A, (PDB #5FH3).

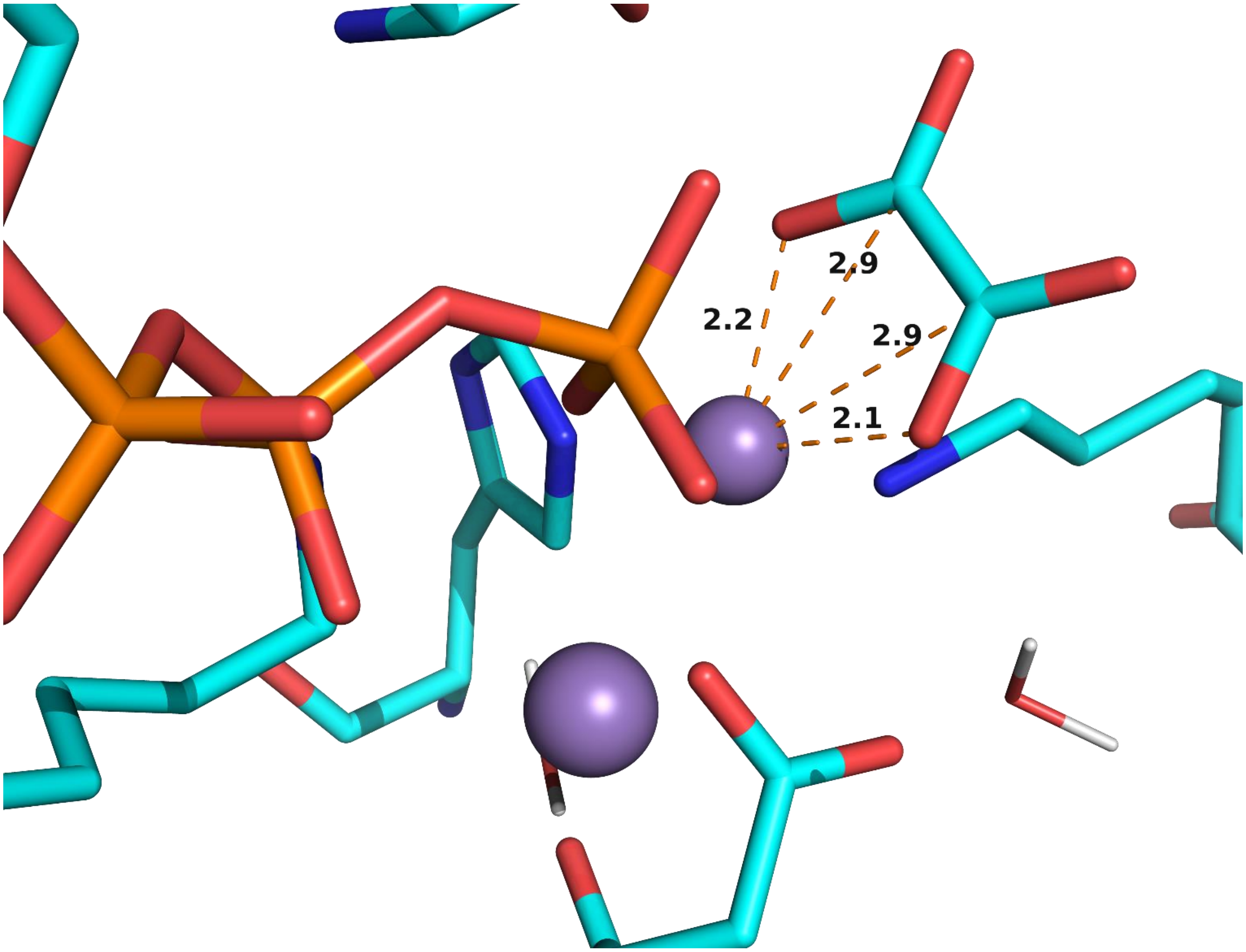

Figure S5q: Mn coordination of oxalate in Rat Cytosolic phosphoenolpyruvate carboxykinase (cPEPCK) Variant H477R, (PDB #5V9G).

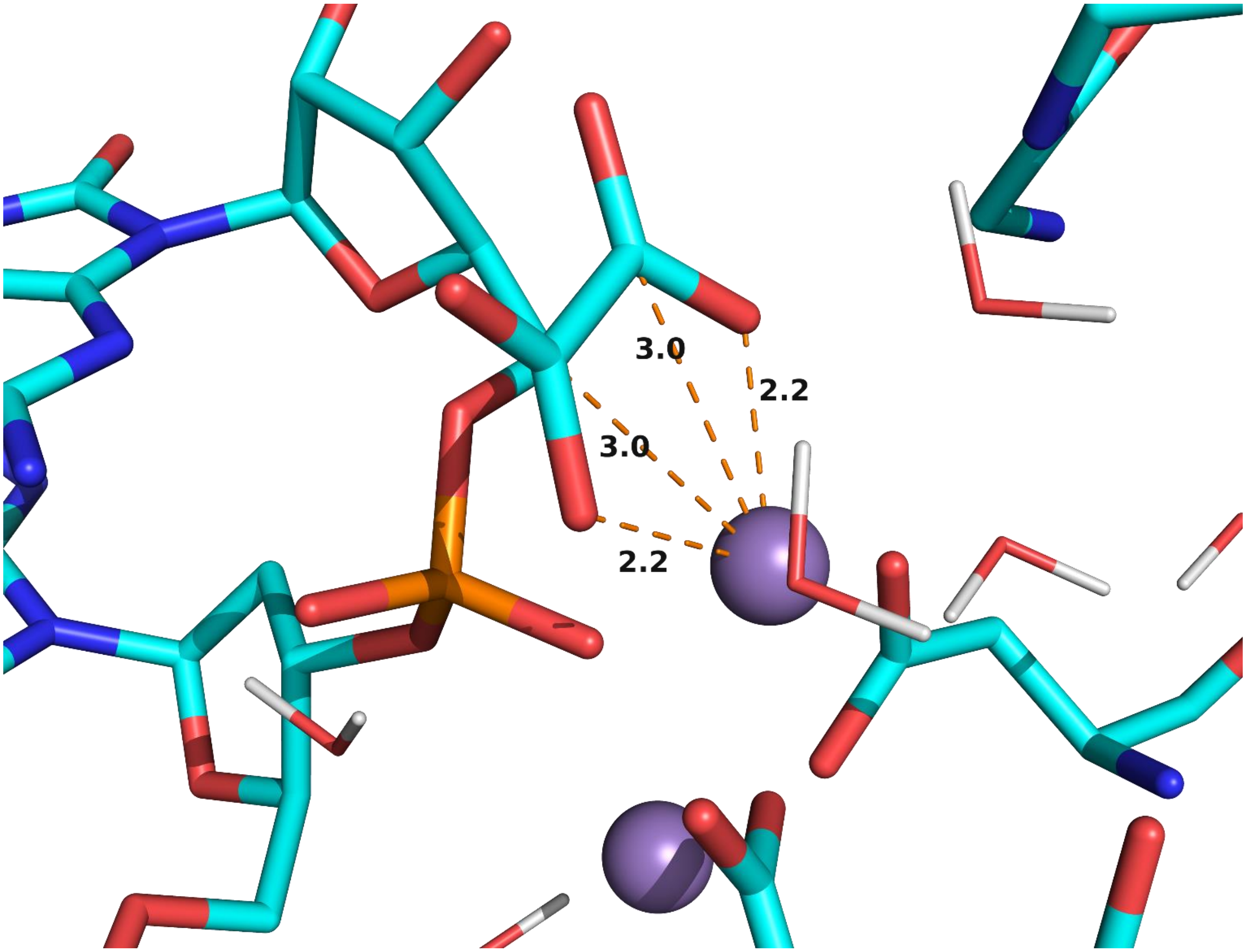

Figure S5r: Mn coordination of oxalate in DNA Polymerase Mu, 8-oxorGTP, (PDB #6VF5).

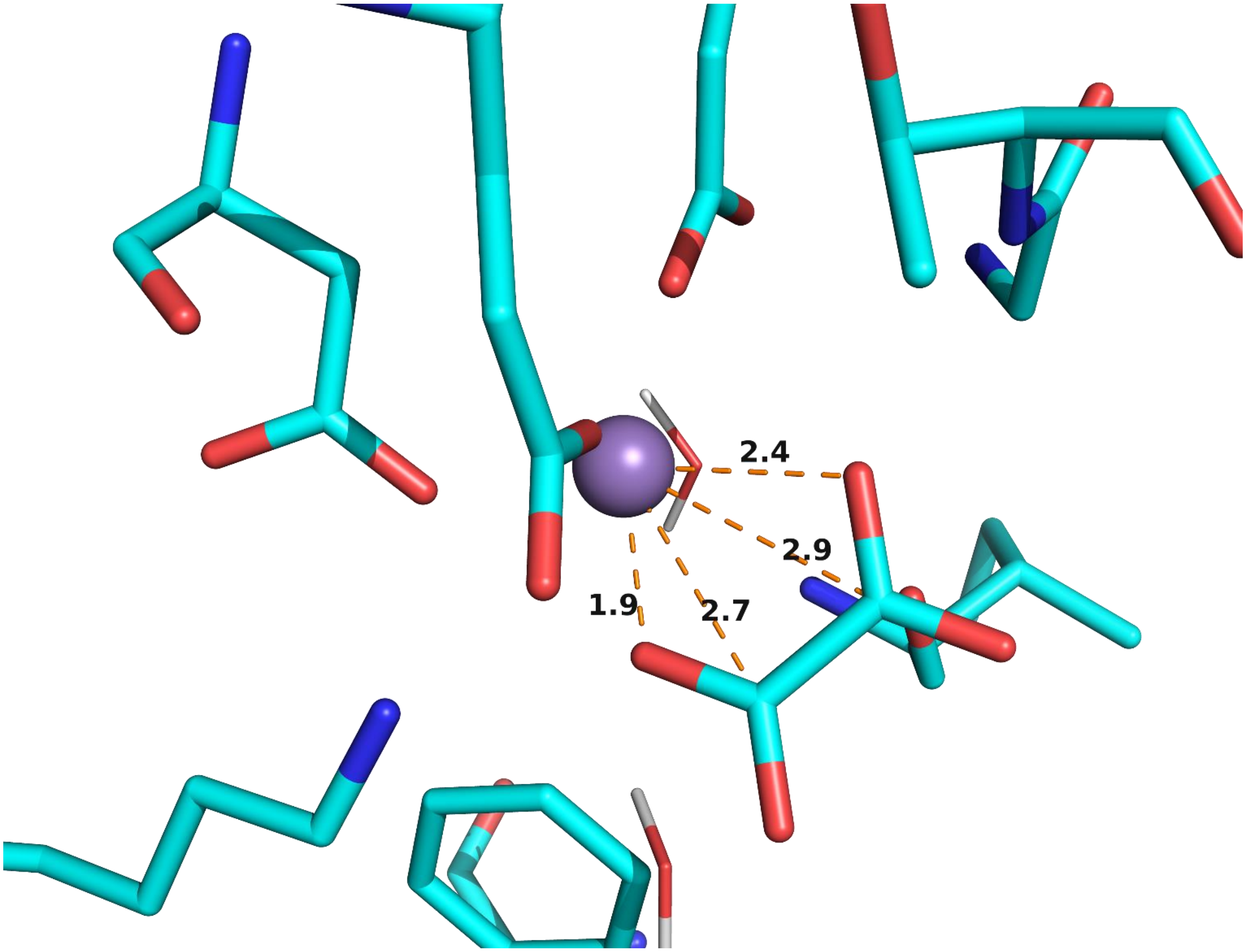

Figure S5s: Mn coordination of oxalate in YisK from Bacillus subtilis, (PDB #8SKY).

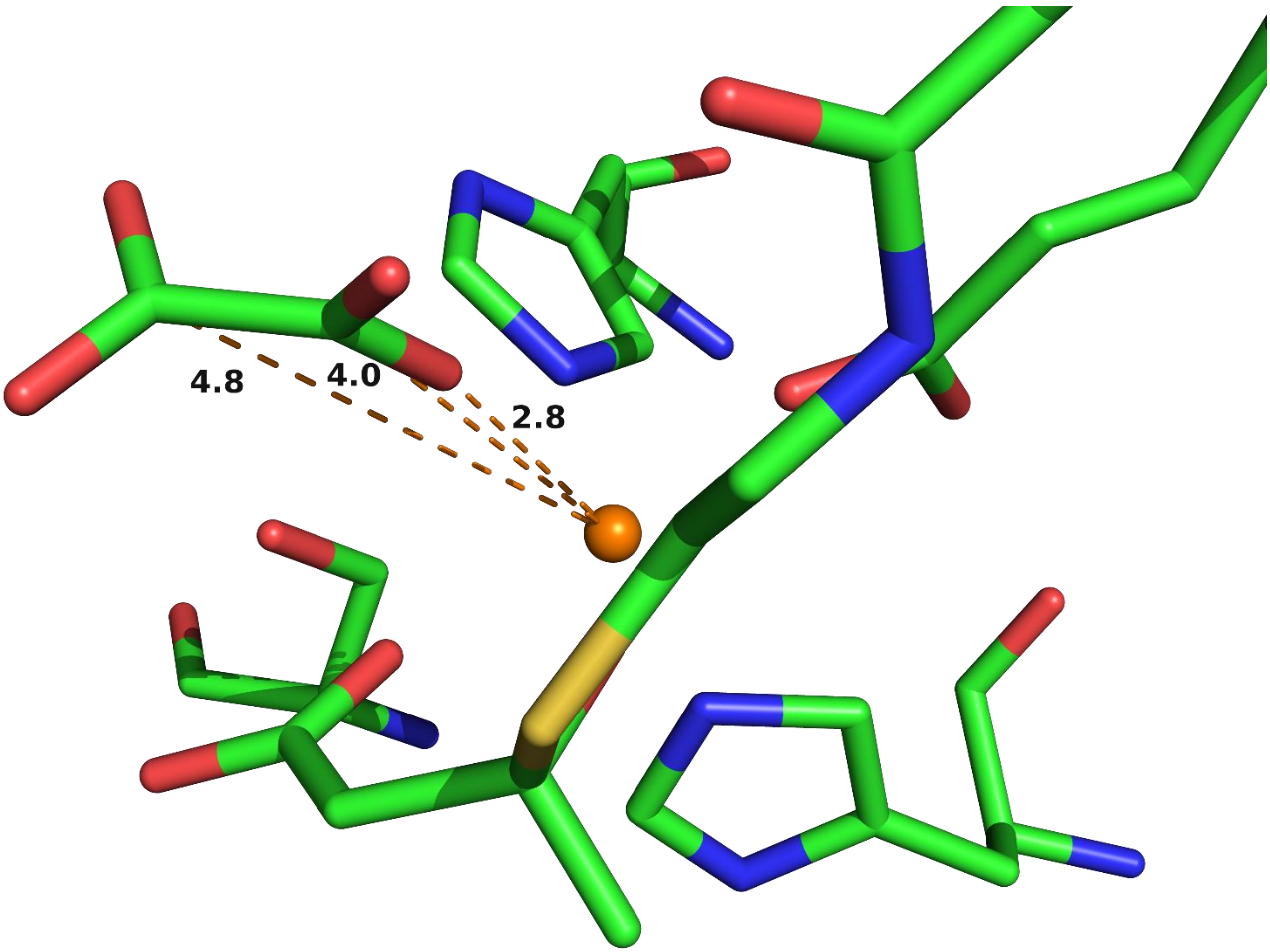

Figure S6a: Co coordination of oxalate in Oxalate Biosynthetic Component A, (PDB #4NNC).

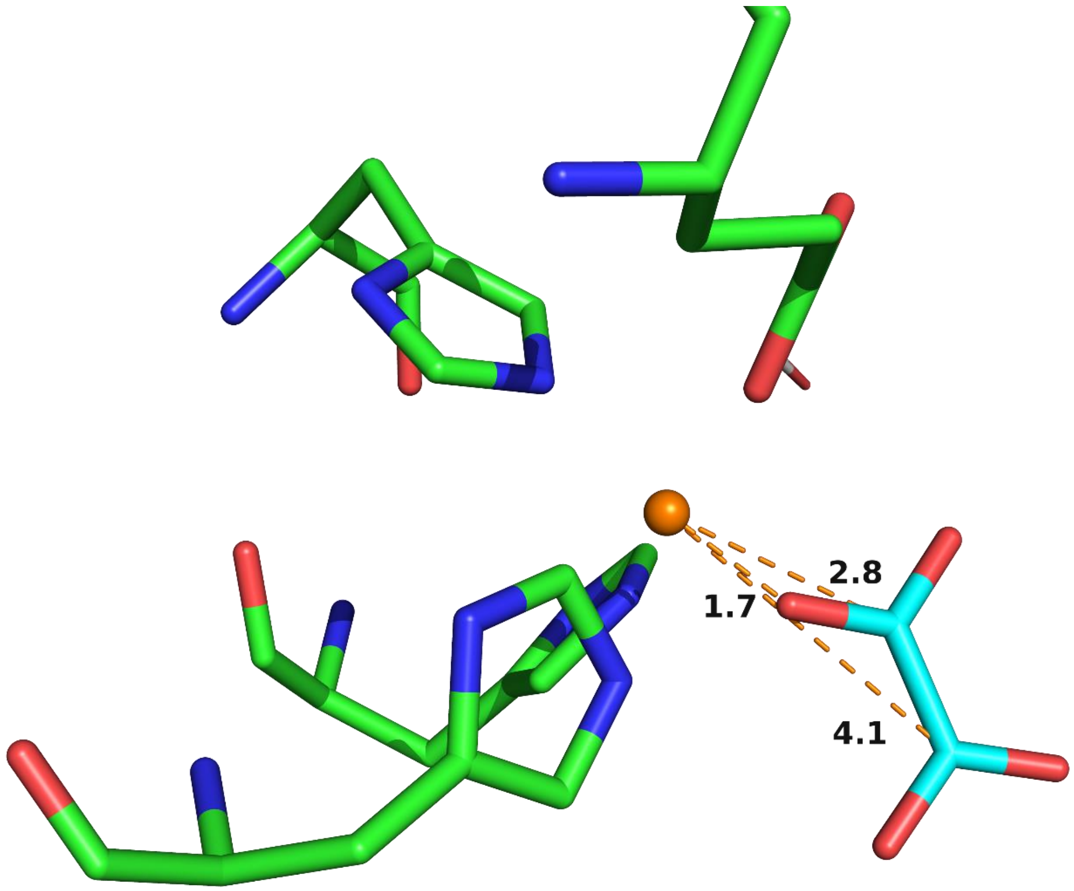

Figure S6b: Co coordination of oxalate in Co-substituted Oxalate Decarboxylase ΔE162, (PDB #5HIO).

Table S1: List of Mn-containing proteins in the PDB database with coordinated oxalate.

PDB ID	Protein Name	# Mn	# OXL	Mn–C distances [Å]	Reference
1DO8	Human Mitochondrial NAD(P)$^+$-Dependent Malic Enzyme	4	4	3.038<br>3.099	[9]
1GQ2	Pigeon Liver Malic Enzyme	16	16	2.934<br>2.946	[10]
1GZ3	Human Mitochondrial NAD(P)$^+$-Dependent Malic Enzyme	4	4	3.056<br>3.075	[10]
1NVM	4-hydroxy-2-ketovalerate aldolase (DmpG)/acylating acetaldehyde dehydrogenase (DmpF)	4	4	2.945<br>2.981	[11]
1O4T	Putative Oxalate Decarboxylase (TM1287) from *Thermotoga maritima*	2	2	2.725<br>2.732	[12]
2RK7	Cytosolic phosphoenolpyruvate carboxykinase (cPEPCK)	4	2	2.978<br>3.019	[13]
3DT2	Cytosolic phosphoenolpyruvate carboxykinase (cPEPCK)	2	1	2.934<br>2.935	[14]
3DT4	Cytosolic phosphoenolpyruvate carboxykinase (cPEPCK)	4	2	2.914<br>2.934	[14]
3M0K	Oxalacetate Acetylhydrolase	1	1	3.034<br>3.050	[15]
3MOF	Cytosolic phosphoenolpyruvate carboxykinase (cPEPCK) mutant A467G	4	2	2.781<br>2.857	[16]
4GMU	Rat Cytosolic phosphoenolpyruvate carboxykinase (cPEPCK) Ld_1g	3	1	2.891<br>2.903	[17]
4GNM	Rat Cytosolic phosphoenolpyruvate carboxykinase (cPEPCK) Ld_2g	2	1	2.760<br>2.849	[17]
4GNQ	Rat Cytosolic phosphoenolpyruvate carboxykinase (cPEPCK) Ld_3g	2	1	2.885<br>2.899	[17]
4JN6	Aldolase-Dehydrogenase Complex from the Cholesterol Degradation Pathway of *Mycobacterium tuberculosis*	2	2	2.862<br>2.959	[18]
4WIU	PEPCK (Rv0211) from *Mycobacterium tuberculosis*	1	1	3.297<br>3.372	[19]
5FH3	Cytosolic PEPCK Variant E89A	2	1	2.703<br>2.849	[20]
5V9G	Rat Cytosolic phosphoenolpyruvate carboxykinase (cPEPCK) Variant H477R	2	1	2.885<br>2.893	[21]
6VF5	DNA Polymerase Mu, 8-oxorGTP	7	1	2.982<br>2.985	[22]
8SKY	YisK from *Bacillus subtilis*	2	2	2.744<br>2.938	[23]

Table S2: List of Co-containing proteins in the PDB database with coordinated oxalate.

PDB ID	Protein Name	# Co	# OXL	Co–C distances [Å]	Reference
4NNC	Oxalate Biosynthetic Component A	1	1	4.019<br>4.830	[24]
5HIO	Co-substituted Oxalate Decarboxylase ΔE162	2	1	2.838<br>4.076	[25]

References

1. Lethbridge, Z. A. D., A. F. Congreve, E. Esslemont, A. M. Z. Slawin, and P. Lightfoot. "Synthesis and structure of three manganese oxalates: $MnC_2O_4 \cdot 2H_2O$, $[C_4H_8(NH_2)_2][Mn_2(C_2O_4)_3]$ and $Mn_2(C_2O_4)(OH)_2$." *Journal of Solid State Chemistry* 172, no. 1 (2003): 212-18.
2. Habjanič, J., M. Jurić, J. Popović, K. Molčanov, and D. Pajić. "A 3D Oxalate-Based Network as a Precursor for the $CoMn_2O_4$ Spinel: Synthesis and Structural and Magnetic Studies." *Inorganic Chemistry* 53, no. 18 (2014): 9633-43.
3. Luan, L. D., J. Li, C. Z. Yin, Z. Lin, and H. Huang. "Solvent-free synthesis of new inorganic-organic hybrid solids with finely tuned manganese oxalate structures." *Dalton Transactions* 44, no. 13 (2015): 5974-77.
4. Puzan, A. N., V. N. Baumer, D. V. Lisovytskiy, and P. V. Mateychenko. "Structure disordering and thermal decomposition of manganese oxalate dihydrate, $MnC_2O_4 \cdot 2H_2O$." *Journal of Solid State Chemistry* 260 (2018): 87-94.
5. Sehimi, H., I. Chérif, and M. F. Zid, "Crystal structure and spectroscopic analysis of a new oxalate-bridged Mn(II) compound: *catena*-poly[guanidinium [[aquachloridomanganese(II)]-μ_2-oxalato-$\kappa^4 O^1, O^2:O^{1'}, O^{2'}$] monohydrate]." *Acta crystallographica. Section E, Crystallographic communications* 72, no. Pt 5 (2016): 724-9.
6. Deyrieux, R., C. Berro, and A. Peneloux. "Contribution à l'étude des oxalates de certains métaux bivalents. III. - Structure cristalline des oxalates dihydratés de manganèse, de cobalt, de nickel et de zinc. Polymorphisme des oxalates dihydratés de cobalt et de nickel." *Bulletin De La Société Chimique De France*, no. 1 (1973): 25-34.
7. Siems, H., and J. Löhn. "Die Kristallstruktur von $Cs_2Mn_2(C_2O_4)_3 \cdot 3H_2O$." *Zeitschrift für anorganische und allgemeine Chemie* 393, no. 2 (1972): 97-104.
8. Chovancova, E., A. Pavelka, P. Benes, O. Strnad, J. Brezovsky, B. Kozlikova, A. Gora, V. Sustr, M. Klvana, P. Medek, L. Biedermannova, J. Sochor, and J. Damborsky. "CAVER 3.0: A Tool for the Analysis of Transport Pathways in Dynamic Protein Structures." *Plos Computational Biology* 8, no. 10 (2012): 12.
9. Yang, Z. R., D. L. Floyd, G. Loeber, and L. Tong. "Structure of a closed form of human malic enzyme and implications for catalytic mechanism." *Nature Structural Biology* 7, no. 3 (2000): 251-57.
10. Yang, Z., C. W. Lanks, and L. Tong. "Molecular mechanism for the regulation of human mitochondrial NAD(P)+-dependent malic enzyme by ATP and fumarate." *Structure* 10, no. 7 (2002): 951-60.
11. Manjasetty, B. A., J. Powlowski, and A. Vrielink. "Crvstal structure of a bifunctional aldolase-dehydrogenase: Sequestering a reactive and volatile intermediate." *Proceedings of the National Academy of Sciences of the United States of America* 100, no. 12 (2003): 6992-97.
12. Schwarzenbacher, R., F. von Delft, L. Jaroszewski, P. Abdubek, E. Ambing, T. Biorac, L. S. Brinen, J. M. Canaves, J. Cambell, H. J. Chiu, X. Dai, A. M. Deacon, M. DiDonato, M. A. Elsliger, S. Eshagi, R. Floyd, A. Godzik, C. Grittini, S. K. Grzechnik, E. Hampton, C. Karlak, H. E. Klock, E. Koesema, J. S. Kovarik, A. Kreusch, P. Kuhn, S. A. Lesley, I. Levin, D. McMullan, T. M. McPhillips, M. D. Miller, A. Morse, K. Moy, J. Ouyang, R. Page, K. Quijano, A. Robb, G. Spraggon, R. C. Stevens, H. van den Bedem, J. Velasquez, J. Vincent, X. Wang, B. West, G. Wolf, Q. Xu, K. O. Hodgson, J. Wooley, and I. A. Wilson. "Crystal structure of a putative oxalate decarboxylase (TM1287) from *Thermotoga maritima* at 1.95 A resolution." *Proteins* 56, no. 2 (2004): 392-5.

13. Stiffin, R. M., S. M. Sullivan, G. M. Carlson, and T. Holyoak. "Differential inhibition of cytosolic PEPCK by substrate analogues. Kinetic and structural characterization of inhibitor recognition." *Biochemistry* 47, no. 7 (2008): 2099-109.
14. Sullivan, S. M., and T. Holyoak. "Enzymes with lid-gated active sites must operate by an induced fit mechanism instead of conformational selection." *Proceedings of the National Academy of Sciences of the United States of America* 105, no. 37 (2008): 13829-34.
15. Chen, C., Q. H. Sun, B. Narayanan, D. L. Nuss, and O. Herzberg. "Structure of Oxalacetate Acetylhydrolase, a Virulence Factor of the Chestnut Blight Fungus." *Journal of Biological Chemistry* 285, no. 34 (2010): 26685-96.
16. Johnson, T. A., and T. Holyoak. "Increasing the Conformational Entropy of the Omega-Loop Lid Domain in Phosphoenolpyruvate Carboxykinase Impairs Catalysis and Decreases Catalytic Fidelity." *Biochemistry* 49, no. 25 (2010): 5176-87.
17. Johnson, Troy A., and Todd Holyoak. "The Ω-Loop Lid Domain of Phosphoenolpyruvate Carboxykinase Is Essential for Catalytic Function." *Biochemistry* 51, no. 47 (2012): 9547-59.
18. Carere, J., S. E. McKenna, M. S. Kimber, and S. Y. K. Seah. "Characterization of an Aldolase-Dehydrogenase Complex from the Cholesterol Degradation Pathway of *Mycobacterium tuberculosis*." *Biochemistry* 52, no. 20 (2013): 3502-11.
19. Kim, H.L., and J.C. Sacchettini. "RCSB PDB - 4WIU: Crystal Structure of PEPCK (Rv0211) from *Mycobacterium tuberculosis* in complex with oxalate and Mn^{2+}." RCSB PDB Protein Data Bank, 2014.
20. Johnson, T. A., M. J. McLeod, and T. Holyoak. "Utilization of Substrate Intrinsic Binding Energy for Conformational Change and Catalytic Function in Phosphoenolpyruvate Carboxykinase." *Biochemistry* 55, no. 3 (2016): 575-87.
21. Cui, D. S., A. Broom, M. J. McLeod, E. M. Meiering, and T. Holyoak. "Asymmetric Anchoring Is Required for Efficient Omega-Loop Opening and Closing in Cytosolic Phosphoenolpyruvate Carboxykinase." *Biochemistry* 56, no. 15 (2017): 2106-15.
22. Jamsen, J.A., A. Sassa, D.D. Shock, W.A. Beard, and S.H. Wilson. "RCSB PDB - 6VF5: DNA Polymerase Mu, 8-oxorGTP:At Product State Ternary Complex, 50 mM Mn2+ (120 min)." RCSB PDB Protein Data Bank, 2021.
23. Guo, T. F., A. M. Sperber, I. V. Krieger, Y. Duan, V. R. Chemelewski, J. C. Sacchettini, and J. K. Herman. "*Bacillus subtilis* YisK possesses oxaloacetate decarboxylase activity and exhibits Mbl-dependent localization." *Journal of Bacteriology* 206, no. 1 (2024): 20.
24. Oh, J., E. Goo, I. Hwang, and S. Rhee. "Structural basis for bacterial quorum sensing-mediated oxalogenesis." *J Biol Chem* 289, no. 16 (2014): 11465-75.
25. Zhu, W., L. M. Easthon, L. A. Reinhard, C. K. Tu, S. E. Cohen, D. N. Silverman, K. N. Allen, and N. G. J. Richards. "Substrate Binding Mode and Molecular Basis of a Specificity Switch in Oxalate Decarboxylase." *Biochemistry* 55, no. 14 (2016): 2163-73.
